# The Photosensitivity of Rhodopsin Bleaching and Light-Induced Increases of Fundus Reflectance in Mice Measured In Vivo With Scanning Laser Ophthalmoscopy

**DOI:** 10.1167/iovs.16-19393

**Published:** 2016-07-12

**Authors:** Pengfei Zhang, Mayank Goswami, Robert J. Zawadzki, Edward N. Pugh

**Affiliations:** 1 Research Investments in Science and Engineering EyePod Small Animal Imaging Facility, University of California-Davis, Davis, California, United States; 2 Department of Ophthalmology and Vision Science, University of California-Davis, Davis, California, United States; 3 Departments of Physiology and Membrane Biology and of Cell Biology and Human Anatomy, University of California-Davis, Davis, California, United States

**Keywords:** rhodopsin bleaching, fundus reflectometry, near infrared scattering

## Abstract

**Purpose:**

To quantify bleaching-induced changes in fundus reflectance in the mouse retina.

**Methods:**

Light reflected from the fundus of albino (Balb/c) and pigmented (C57Bl/6J) mice was measured with a multichannel scanning laser ophthalmoscopy optical coherence tomography (SLO-OCT) optical system. Serial scanning of small retinal regions was used for bleaching rhodopsin and measuring reflectance changes.

**Results:**

Serial scanning generated a saturating reflectance increase centered at 501 nm with a photosensitivity of 1.4 × 10^−8^ per molecule μm^2^ in both strains, 2-fold higher than expected were irradiance at the rod outer segment base equal to that at the retinal surface. The action spectrum of the reflectance increase corresponds to the absorption spectrum of mouse rhodopsin in situ. Spectra obtained before and after bleaching were fitted with a model of fundus reflectance, quantifying contributions from loss of rhodopsin absorption with bleaching, absorption by oxygenated hemoglobin (HbO_2_) in the choroid (Balb/c), and absorption by melanin (C57Bl/6J). Both mouse strains exhibited light-induced broadband reflectance changes explained as bleaching-induced reflectivity increases at photoreceptor inner segment/outer segment (IS/OS) junctions and OS tips.

**Conclusions:**

The elevated photosensitivity of rhodopsin bleaching in vivo is explained by waveguide condensing of light in propagation from rod inner segment (RIS) to rod outer segment (ROS). The similar photosensitivity of rhodopsin in the two strains reveals that little light backscattered from the sclera can enter the ROS. The bleaching-induced increases in reflectance at the IS/OS junctions and OS tips resemble results previously reported in human cones, but are ascribed to rods due to their 30/1 predominance over cones in mice and to the relatively minor amount of cone M-opsin in the regions scanned.

Rods are the most numerous cell type in human and mouse retinas, and rhodopsin, the visual pigment of rods, is the most abundant retinal membrane protein. Mutations in rhodopsin are a major cause of retinal degeneration, typically autosomal dominant retinitis pigmentosa.^1–3^ Because of the pivotal roles of rhodopsin and of the visual retinoid cycle in ocular health and disease, measurement of rhodopsin in the living eye is a valuable assay in basic and clinical visual science.

Rhodopsin has been investigated in the living human eye for over 60 years with fundus reflectometry,^4–7,8^ providing much insight into basic mechanisms and disease (reviewed in Refs. 9–11). In contrast, while the bleaching and regeneration of mouse rhodopsin in situ have been quantified with ex vivo spectroscopy,^12–14^ to our knowledge no in vivo measurements of rhodopsin in mice with fundus reflectometry have been published. The disparity between the application of fundus reflectometry to human and mouse is due in part to the optical challenges involved in making the measurements in the much smaller mouse eye. The disparity may also be due to difficulties associated with the use of anesthetics and with corneal and lens clouding in mice, and to the availability of ex vivo extraction methods for rhodopsimetry and retinoid analysis in mice. Despite the difficulties, in vivo measurements of rhodopsin in mice would have distinct virtues, including the potential for longitudinal study in individual animals. Instrumentation for imaging the mouse fundus has developed rapidly in the past few years, providing better understanding of the practical issues involved. One such issue is that the adult mouse eye has a much shorter posterior nodal distance (∼2 mm) than the human eye (16.7 mm)^15^; as a consequence, scanning laser ophthalmoscopy (SLO) of a specific solid angle in mice and humans with a light beam of the same power will result in ∼(16.7/2)^2^ = 73-fold greater photon flux density per unit retinal area in the mouse. Thus, the SLO imaging power for making rhodopsin measurements in the mouse needs to be far lower than that used in human experimentation. We have recently constructed a multimodal mouse retinal imaging system that allows simultaneous wide-field SLO and optical coherence tomography (OCT) data acquisition,^16^ and used this system to quantify the minimum light levels needed to obtain good-quality fundus reflectance and autofluorescence images (Zhang P, et al. *IOVS* 2015;56:ARVO E-Abstract 4116). With this custom imaging system we were able to overcome the challenges to measuring rhodopsin and its bleaching in the living mouse eye.

## Methods

### Mouse Strains and Handling

All mouse husbandry and handling were in accord with protocols approved by the University of California Animal Care and Use Committee (IACUC), which strictly adheres to all National Institutes of Health (NIH) guidelines and satisfies the ARVO Statement for the Use of Animals in Ophthalmic and Vision Research. Pigmented C57BL/6J (*n* = 4, 6–7 months old) and albino Balb/c mice (*n* = 7, 3–7 months old) were obtained from Jackson Laboratories (Bar Harbor, ME, USA) at age 2 months and maintained on a 12:12, ∼100-lux light cycle.

### General Protocol

**Figure 1:**
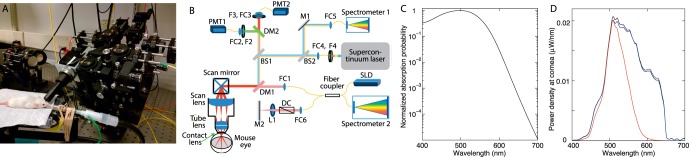
Multimodal SLO/OCT imaging system and key spectral quantities employed in the experiments. (**A**) Picture of the scanning apparatus, with an anesthetized mouse emplaced. (**B**) Optical schematic of the imaging system (not to scale). Abbreviations: BS, beam splitter; DM, dichroic mirror; FC, fiber optic coupler; M, silver mirror; PMT, photomultiplier. (**C**) Normalized absorption spectrum of mouse rhodopsin (*λ*_max_ = 498 nm) generated with the Lamb-Govardovskii template for pigment extinction spectra assuming the axial optical density at the *λ*_max_ to be OD_max_ = 0.35 (right-hand side of Equation 14). (**D**) Spectral power density function of the broadband source (Fianium supercontinuum laser) at the plane of the pupil as reflected by a Fluorilon 99W disc: The *black curve* presents the spectrum uncorrected for the transmission losses between the Fluorilon disc and the spectrometer; the *blue curve* presents the spectrum corrected for the losses (Methods). In both cases the integral of the spectral power density function was equated with the power of the beam at the pupil measured with a Thorlabs radiometer with wavelength set to 580 nm (Supplementary Material II). The *red curve* shows the spectrum weighted by the rhodopsin absorption spectrum given in (**C**) (the spectral density function was converted to photon units before weighting with the template spectrum, and reconverted back to radiometric units).

Mice were dark adapted overnight in a light-proof container with water and food. Under dim red light (80-nm band centered at 655 nm), a dark-adapted mouse was removed from the container, anesthetized with 2% isoflurane delivered in O_2_, and then transferred to a heated, adjustable platform adjacent to the imaging apparatus (Fig. 1A). The mouse's snout was quickly fitted into an anesthetic delivery tube that included a bite bar over which its incisors were hitched, providing for both snug fitting of the tube and rigid control of the mouse's head position. The pupils were then dilated and eyes cyclopleged with drops of tropicamide and phenylephrine, and Gel Tears (Chem-Pharm Fabrik, Berlin, Germany) were applied to maintain a hydrated cornea and provide a minimally refracting interface with a 0-diopter contact lens (Unicon Corp., Osaka, Japan) mounted at the apex of the SLO imaging optics.

### Imaging Apparatus and Fundus Imaging Procedures

The multimodal imaging apparatus comprises an SLO unit and an OCT unit (Fig. 1B) as previously described.^17^ A supercontinuum laser (SC-400; Fianium, Southampton, Hampshire, UK) served as the light source for the SLO subsystem (Fig. 1B). By choice of bandpass filters F2 and F3, light of different wavelengths could be selected for imaging. Reflected light from the retina was acquired by PMT1 for long-wavelength light and PMT2 for mid-wavelength light (H7422-50, -40, respectively; Hammatsu City, Shizuoka, Japan). The reflected light could be also directed into spectrometer1 (QE65000; Ocean Optics, Dunedin, FL, USA) by mirror M1 to allow measurement of reflectance spectra. When used for spectrometry, the SLO can operate either in point excitation mode (no scanning) or region of interest (ROI) mode (with scanning), although the recorded spectral information in the latter mode is collected from the entire ROI. The imaging beams had a diameter of 0.44 mm at the plane of the anterior corneal surface.

The OCT subsystem uses a broadband superluminescent (SLD) light source (Broadlighter, 890; Superlum, Carrigtohill, Ireland) with 132-nm bandwidth centered on 860 nm, and delivers 600 μW at the mouse pupil plane. The theoretical axial resolution of the OCT is ∼2 μm. Further details are provided elsewhere.^17^

#### Light Calibrations.

The radiant power of the imaging light as it emerged from the contact lens at the position of the mouse cornea was measured with a Thorlabs (Newton, NJ, USA) S120C photodiode-based power meter that had been calibrated against an NBS standard. Imaging light power was measured prior to each experiment and adjusted to standard values as described below.

#### SLO Imaging, Retinal Scaling.

**Figure 2:**
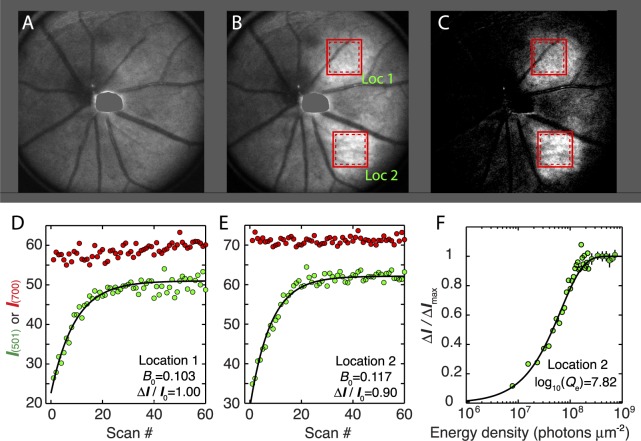
Light-stimulated increase in fundus reflectance for 501-nm light with serial scanning of small retinal areas. (**A**, **B**) Images taken with 501-nm light immediately before (**A**) and after (**B**) the serial scanning of locations 1 and 2. Images in (**A**, **B**) were obtained with the same PMT gain settings and are displayed with identical contrast scaling; each is the average of six full-FOV scans with 1-μW laser power. (**C**) Contrast-adjusted image of the difference between images (A, B) revealing the increased reflectance of the serially scanned regions. (**D**, **E**) The spatially averaged intensity of the 501-nm light reflected from the retinal regions enclosed in the *dashed boxes* is plotted as a function of the scan number (*green symbols*) and fitted by least squares with Equation 11 (*smooth curves*) to estimate the parameters *I*_0_, Δ*I*, and *B*_0._ The average intensities of the simultaneously collected 700-nm light (*offset* for convenience of display on the same plots) are shown as *red symbols*. (**F**) The incremental reflectance data of (**E**) are normalized and replotted as a function of the cumulative energy density. The smooth curve was generated as the complement of Equation 12, that is, 1 − *p_n_* with *Q_e_* = *Q*_1_/*B*_0_ = 10^7.82^ photons μm^−2^ (no free parameters). For the cumulative energy densities in the saturating region of the curve, the points were averaged in bins of 10 and are plotted as mean ± SEM.

The maximum visual angle of the fundus scan of the SLO was measured to be 51°. In converting visual angles to retinal distances, we initially adopted a conversion factor of 34 μm/deg, corresponding to a posterior nodal distance (PND) of 1.95 mm.^15^ However, as the contact lens causes the nodal point of the combined contact lens and mouse eye optical system to shift to a position more anterior than the nodal point of the eye alone, we compared overlaid SLO fundus images (centered on the optic nerve) of the retinal vasculature with flat-mount explants of the retinas of the same eyes (Supplementary Material I; Fig. 2A). We determined the calibration factor with the contact lens to be 43 μm/deg, a 26% linear expansion of the image field over that calculated with the standard PND. The standard *x*-*y* scan mapped to 256 × 256 pixels, with a sampling duration of 6 μs/pixel and a total measured light exposure duration of 0.461 second. The latter value includes 17.2% “flyback” time during which the galvanometer mirror was returning to its initial scan position while scanning back across the tissue. Initial alignment of the dark-adapted mouse was performed with near-infrared (NIR) light (700 ± 20 nm, 5 μW). Before and after each experiment a reference SLO “background” image was recorded for each bleaching location with the imaging/bleaching lights on, but without the mouse present in the imaging system. These reference data were subtracted from the experimental data obtained with the mouse in place to ensure that no light backscattered from the optical elements of the imaging system affected the retina reflectance data.

#### Bleaching Protocol.

Bleaching was accomplished by repeated scanning of a standard small field of view (FOV) of 9° × 9° (387 × 387 μm), usually 100 times. Simultaneous dual-band scanning was performed with a mid-wavelength light (468, 501, or 542 nm; 10-nm bandpass) and with a deep red, long-wavelength (700 nm) light. Mid-wavelength imaging light power entering the mouse pupil was always set to 1 μW, while the power of the 700-nm beam was set to 5 μW. Calculations with a mouse rhodopsin template spectrum (Fig. 1C) showed that the 5 μW 700-nm light would bleach rhodopsin at a 5.5 × 10^−5^ lower rate per scan than the 1 μW 501-nm light (Fig. 1C).

#### Broadband Bleaching and Reflectance Measurements.

In some experiments bleaching was performed with broadband laser light (Fig. 1D), and reflected light captured by the spectrometer was used to measure spectral reflectance changes accompanying bleaching exposures. In these experiments the total power of the light entering the mouse pupil was set to ∼2 μW, measured with the Thorlabs S120C photodiode with its wavelength setting at 580 nm. The spectral power distribution function (W/nm) of the light at the pupil was determined from the spectrum of a model eye created with a Fluorilon 99W reflecting disc (Avian Tech, Gainesville, FL, USA) at the “retinal” plane; the Fluorilon disc acted as a Lambertian reflector with extremely flat (<0.5% deviation) reflectance spectrum. The transmission characteristics of the optical elements were taken into consideration (Fig. 1D; Supplementary Material II). For analysis of bleaching, the spectral energy density was converted into rhodopsin-equivalent units, using a Lamb^18^-Govardovskii^19^ template for mouse rhodopsin, assuming the axial density of rhodopsin in mouse rods to be 0.35 (Figs. 1C, 1D; Supplementary Material II).

### Analysis of Reflectance Spectra and Bleaching Difference Spectroscopy

The use of a broadband supercontinuum light source and the inclusion of a spectrometer as a detector in the SLO afforded the opportunity of measuring bleaching-induced changes in reflectance across the whole visible spectrum. Defining *P_in_*(*λ*) as the spectral distribution of the laser power (W/nm) of the light entering the eye, and *P_out_*(*λ*) as the power of the light reflected from the fundus and collected by the spectrometer, the ocular reflectance spectrum *ρ*(*λ*) is defined by

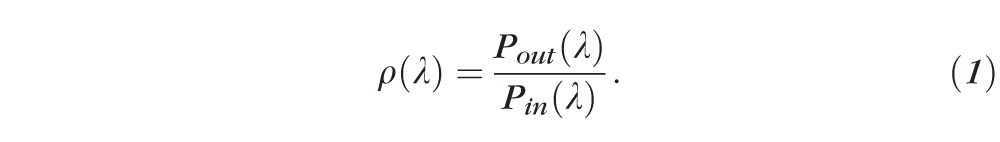



In practice, *P_in_*(*λ*) could not be directly measured in absolute units with light back-reflected from the corneal plane due to use of a confocal aperture in the system, as well as to loss in the fiber optic coupling of the spectrometer input. To extract a spectrum proportional to *P_in_*(*λ*) we employed the model eye, as described above (Fig. 1D). The absolute spectral distribution was determined by equating the measured total power with the numerically integrated spectrum measured with the spectrometer (Supplementary Material II).

Measurement of the absolute spectrum of light reflected from the eye, *P_out_*(*λ*) (Equation 1), was achieved as follows. First, absolute calibration of the Ocean Optics 65000 spectrometer was made using two approaches: direct measurement of broadband light filtered with a series of nine narrowband filters (located at F4 in Fig. 1B) with midpoints ranging from 441 to 646 nm, and calculation from the manufacturer's specifications. The first approach resulted in a calibration factor of (4.48 ± 0.80) × 10^−17^ J/nm/count (mean ± SD, *n* = 9) while the second gave (3.00 ± 0.63) × 10^−17^ J/nm/count. In the first approach the power of each narrowband source was measured with the Thorlabs power meter as it exited from the fiber optic that normally delivered the light to the spectrometer. We elected to use the directly measured calibration factor, as the manufacturer specifications from which the second estimate was derived were provided only as an average value in the company literature. We next determined the total fractional transmission through the optics between the contact lens and the spectrometer to be 0.00825; this latter factor includes a measured 10% transmission efficiency in the fiber optic coupler to the spectrometer (FC5 in Fig. 1B). With these factors in hand, we converted the spectra of light reflected from the mouse eye and captured by the spectrometer into absolute power units “at the cornea.”

In using the spectrometer, our goal was to measure ocular reflectance spectra as defined by Equation 1. From inspection of Figure 1B, it can be seen that most of the optical elements—including the antireflection coatings of the lenses, the dichroic mirror DM1, and the beamsplitter BS1—are shared by the input and output paths. Thus, any spectral distortions that these optical elements might produce would tend to be cancelled out in the ratio (Equation 1). Moreover, analysis with optical specifications predicted spectral distortion to be very small (Fig. 1D). Thus, we are confident that (subject to the signal/noise limits of the measurements) the reflectance spectra were not distorted.

Bleaching difference spectra were measured by repeatedly scanning a standard small FOV (387 × 387 μm) with the broadband source, using the protocol described above for narrowband light, but directing all light reflected from the retina to the spectrometer. The *n*th (*n >* 1) difference spectrum *BD_n_*(*λ*) is defined as

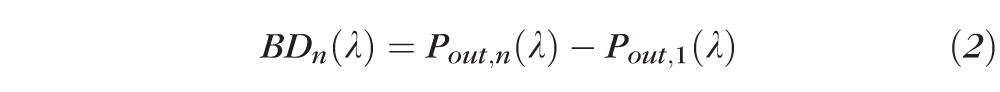
where *P_out,n_*(*λ*) is the spectrum measured during the *n*th scanning cycle.

### Rhodopsin Bleaching When Pigment Is Present “In Density”

A primary goal of this work is the quantification of rhodopsin bleaching in mice in vivo. Microspectrophometry^20–22^ and spectroscopic measurement of extracted rhodopsin^23^ have established that the specific axial density of rhodopsin in rod outer segments is 0.014 to 0.018 OD (optical density)/μm. It follows that a mouse rod outer segment of average length 22 μm^24^ will have an OD_max_ of 0.31 to 0.40 for axially propagating light of the wavelength of mouse rhodopsin's *λ*_max_ (498 nm). Such an OD necessarily causes “self-screening,” a decrease in the power with axial depth in the dark-adapted outer segment, and under such conditions the pigment is said to be present “in density.” The rate equation governing rhodopsin bleaching in the absence of regeneration when pigment is present in density and the retina is exposed to light having energy density *Q*(*λ*) (unit: photons μm^−2^) is expressible as follows^4,8^ :

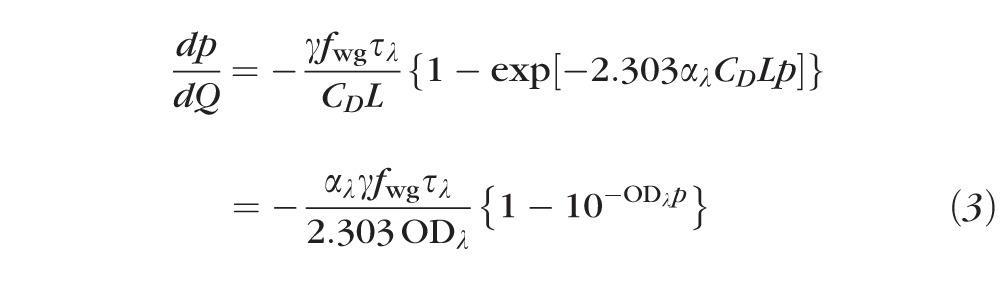



Here *p* is the fraction of rhodopsin present, *τ_λ_* is the transmissivity of the ocular media and retina up to the depth where light is coupled into the rod inner segment waveguide, *f*_wg_ is a dimensionless factor (≥1) accounting for the effect of the waveguiding in “condensing” of light from the larger-diameter inner segment to the smaller-diameter outer segment, and *α_λ_γ* is the intrinsic photosensitivity for axially propagating light of wavelength *λ*, with *α_λ_* (unit: μm^2^ molecule^−1^) the extinction coefficient of rhodopsin in situ and *γ* the quantum efficiency of isomerization/bleaching,^25,26^ *C_D_* the concentration of rhodopsin in the dark-adapted rod, and *L* the length of the outer segment (expressed in units compatible with *α_λ_*). The exponential term in the curly brackets in Equation 3 arises from Beer's Law and represents the probability that a photon is captured in traversing the outer segment, while the constants preceding this term scale the light energy density delivered to the outer segment (*τ_λ_*_,_ *f*_wg _), give probability that a captured photon isomerizes rhodopsin (*γ*), or serve to render the units consistent (*p* = *C*/*C_D_*). The second line of Equation 3 reformulates the first line in terms of the axial OD*_λ_*.

Equation 3 can be integrated analytically, but the result is an implicit equation in *p*, that is, one in which *p* is present in more than one term and cannot be separated. The implicit equation, however, can readily be solved by numerical methods to obtain the dependence of *p* on *Q* to generate “fraction bleached versus energy density” curves. When OD_max_ is less than approximately 0.1, Equation 3 is well approximated by

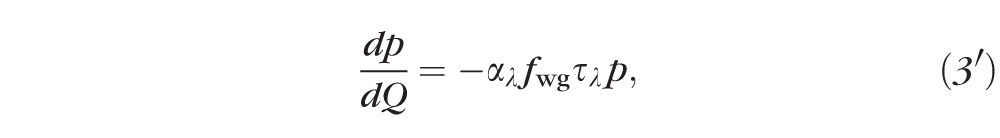

,
which has the simple solution

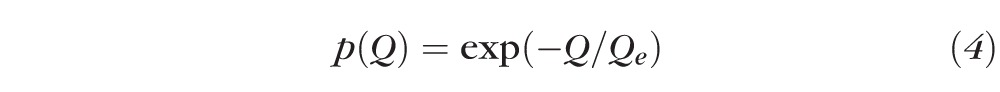
with

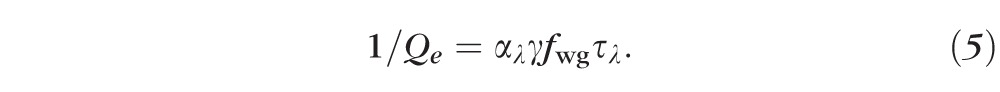



Equation 4 has classically been used to characterize the light dependence of bleaching data in human fundus reflectometry.^7,27,28^ Experimentally, *Q_e_* is the empirically measured energy density that reduces the fraction rhodopsin present to 1/e of its dark-adapted level, while 1/*Q_e_* is the apparent “photosensitivity of rhodopsin in situ” for light measured at the cornea but expressed in energy density at the retina. The intrinsic photosensitivity (*α_λ_γ*) can be thought of as the apparent cross-sectional area of a rhodopsin molecule in situ for capturing a photon of a specific wavelength (*α_λ_*) multiplied by the probability or quantum efficiency (*γ*) that a captured photon leads to the isomerization of the 11-*cis* retinal chromophore.

Equation 4 is much more convenient for quantifying the dependence of rhodopsin bleaching on energy density (e.g., in least-squares fitting) than solutions to Equation 3. However, as it is incontrovertible at this point in the history of retinal research that rhodopsin is present in density in rods, it is important to assess the degree to which use of Equation 4 to describe bleaching data will bias estimation of the photosensitivity. To address this issue, in Supplementary Material III solutions to Equation 3 are presented for two cases, OD_max_ = 0.35 (the value assumed throughout this paper) and OD_max_ → 0, or “low density” (Supplementary Fig. S2>). The form of the solution of Equation 3 for OD_max_ = 0.35 and the low density case are very similar, as seen by shifting the solution for OD_max_ = 0.35 to the left by 0.107 log_10_ units (i.e., a factor of 1.28) on the intensity axis (Supplementary Fig. S2, red curve). The net result of these considerations is that for OD_max_ = 0.35, use of Equation 4 will cause the estimate of *Q_e_* to be 1.28-fold higher and photosensitivity to be 1.28-fold lower than the values obtained by fitting bleaching data with the solution of Equation 3 (Supplementary Material III). More generally, an appropriate correction factor can be derived for any specific value of OD_max_. Note that the interpretation of 1/*Q_e_* (Equation 5) is the same regardless of whether Equation 4 or the solution of Equation 3 is applied to bleaching data: What is dependent on the value of OD*_λ_* is the fractional bleach produced by light of energy density *Q_e_* (Supplementary Material III).

### Analysis of Rhodopsin Bleaching by Dual-Wavelength SLO Scanning

Bleaching with serially repeated scanning admits a straightforward analysis of the bleaching rate. Thus, if each small-FOV scan bleaches a fraction *B*_0_ of the rhodopsin present in the scanned region, then the fraction *p_n_* of rhodopsin present when photons arrive during the *n*th scan will be given by

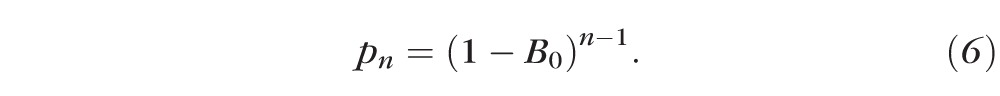



The reason that *n* − 1 appears in the exponent of Equation 6 is that the scanning beam sweeps across the retina at 6 μs/pixel and bleaching—the photoisomerization-induced transition of rhodopsin to metarhodopsin II—takes ∼1 ms. It follows that fundus reflection increases caused by loss of rhodopsin absorption are delayed in appearance by one scan (500 ms) from the scan in which photoisomerization occurs. In brief, though the 1-ms delay between rhodopsin photoisomerization and subsequent bleaching is not usually important, it is material for the analysis of bleaching data in the present experiments.

The dual-band SLO scanning with *λ*_1_ = 501 nm and *λ*_2_ = 700 nm provides two simultaneously collected, time-varying signals

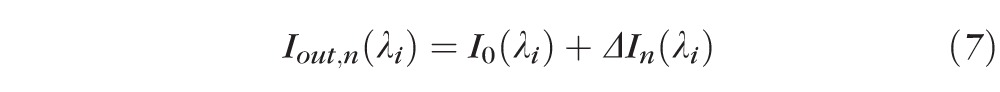
where *I*_*out*_*_,n_*(*λ_i_*), the average intensity of the light reflected from the small FOV during the *n*th scan, is separated into two components: a quantity *I*_0_ representing the light reflected from the fundus before any rhodopsin is bleached, and an incrementing quantity *ΔI_n_* that reaches a maximum when rhodopsin is fully bleached. In some experiments we observed an increase in reflected light in the 700-nm channel. Because rhodopsin absorbs negligibly at 700 nm (Fig. 1C), this change must arise from a bleaching-induced increase in reflectance, and not merely from the loss of rhodopsin absorption per se. We hypothesize (and present evidence to support the view) that the underlying increase in reflectivity occurs across the visible and NIR spectrum, and we further propose a correction factor that assumes that the spectral dependence is flat. Specifically, we propose that the incremental reflected light in the 501-nm channel can be separated into two components, one arising from loss of rhodopsin absorption and a second from the increased backscattering:

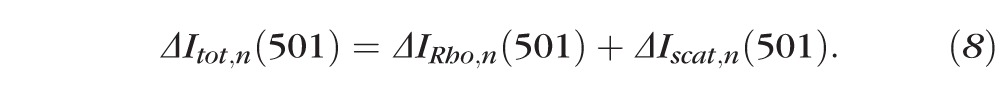



The hypothesis that the increased backscattering is spectrally flat is expressed by

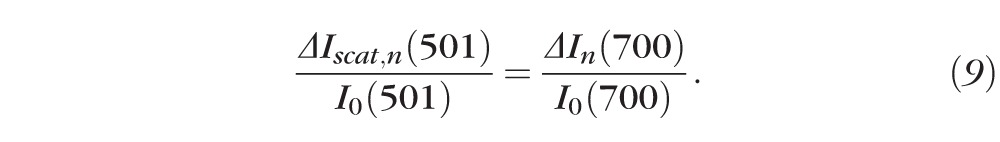



To obtain a signal that should arise solely from the loss of absorption by rhodopsin, when there was an increase in 700-nm reflectance accompanying bleaching exposures, the 501-nm reflected light was adjusted by

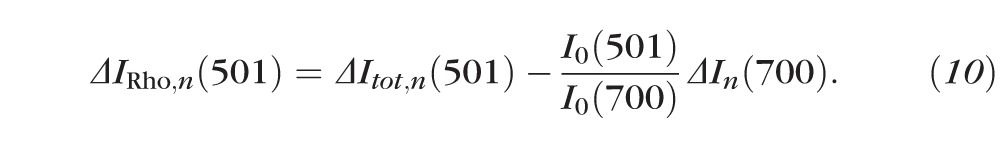



When needed, adjustments for scattering with Equation 10 were made by first fitting a smooth function to the NIR scattering series Δ*I_n_*(700) and using the smooth function in lieu of the noisier trial-by-trial measurements. Given that pigment bleaches in serial scanning according to Equation 3, the “rhodopsin” signal extracted with Equation 7 should then obey the relation

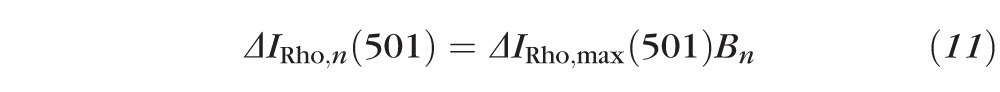
where *B_n_* = 1 − *p_n_* is the fraction of rhodopsin in the bleached state during the *n*th scan (Equation 6). We used a custom Matlab (Natick, MA, USA) script to fit Equation 7 by least-squares minimization to bleaching series data, with *I_out,n_* measured as the average “pixel intensity” of *n*th SLO frame, and *I*_0_, Δ*I*_max_, and *B*_0_ the parameters estimated by fitting (we will suppress the wavelength dependence in Equation 7 when it is clear that we are referring to the 501-nm reflected light).

The dependence of bleaching on energy density during SLO scanning can be quantified by treating the retina as though it were exposed to a spatially homogenous step of light in which each scan delivers a time-averaged retinal irradiance *Q*_1._ The value of the latter was determined from measured factors as *Q*_1_ = (*P_in_*(*λ*)Δ*T*)/*A*_bleach_, where *P_in_*(*λ*) is the laser power at the cornea (units: photons s^−1^), Δ*T* the scan duration (unit: s), and *A*_bleach_ (unit: μm^2^) the area of the retina scanned; note that *Q*_1_ has the units of energy density, that is, photons μm^−2^. Thus, a variant of Equation 4 appropriate for bleaching by serial scanning is

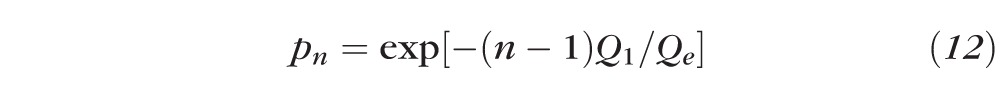
where the total energy density of light *Q* delivered prior to the *n*th scan is *Q* = (*n* − 1)*Q*_1_. It is useful to note that Equations 6 and 12 are closely related, because for small trial-by-trial fraction bleached (*B*_0_), the geometric decline function (Equation 6) is closely approximated by an exponential decay (Equation 12). As a consequence, when the scan-by-scan increases in reflectance during serial bleaching are describable by Equations 7 and 11, then Equation 12 will predictably describe the dependence of the incremental reflectance on the cumulative irradiance delivered by the serial scanning. Moreover, for quantitative consistency between Equations 6 and 12, when each scan bleaches a small fraction (<15%) of rhodopsin, the right-hand side of Equation 12 with *n* = 2 is well approximated by its first McLaurin series expansion term as 1 − *Q*_1_/*Q_e_*, and so *B*_0_ = 1 − *p*_2_ = *Q*_1_/*Q_e_*. Thus, estimating *B*_0_ by least-squares fitting of Equation 7 to serial bleaching data automatically yields the estimate *Q_e._* = *Q*_1_/*B*_0_, where *Q*_1_ is the energy density of a single scan (expressed in photons μm^−2^). In summary, for each set of serial scan data we estimated *B*_0_ by application of Equations 6–11, and then derived *Q_e_* from the relation *Q_e._* = *Q*_1_/*B*_0_.

### Predictions Based on Fundamental Properties of Mouse Rhodopsin In Situ

#### Rhodopsin Absorption Spectrum.

An action spectrum is the spectral energy or power distribution of a set of monochromatic lights that each produce the same, constant effect. For the present experiments, we select the constant effect to be a specific magnitude increase in fundus reflectance. Because the dependence of incremental reflectance on retinal energy density *Q*(*λ*) is well described as an exponential rise Δ*I*/Δ*I*_max_ = 1 − exp[−*Q*(*λ*)/*Q_e_*(*λ*)], then the hypothesis that bleaching of rhodopsin underlies the increased reflectance predicts the measured energy density *Q_e_*(*λ*) corresponding to a constant number of isomerizations (bleached rhodopsins) per rod, which can be expressed as

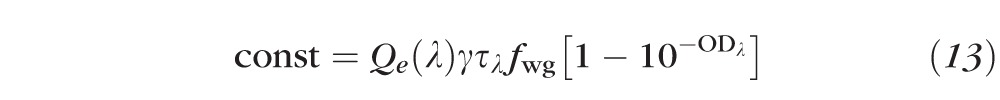
or, rearranging terms, as

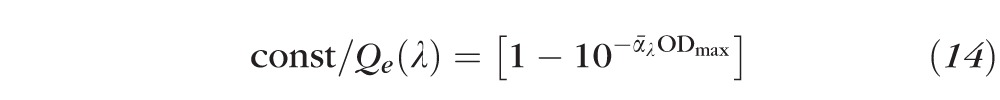
where Equation 14 reparameterizes Equation 13 in terms of the normalized extinction spectrum *ᾱ_λ_* = *α_λ_*/*α_λ_*_max_, and OD_max_ is the end-on OD of rhodopsin in dark-adapted rods at the *λ*_max,_ 498 nm. Thus, the measured action spectrum *Q_e_*(*λ*) of reflectance increase is predicted to be inversely proportional to the absorption spectrum of rhodopsin in situ. The right-hand side of Equation 14, uncorrected for media transmission and waveguide factors (i.e., with *f*_wg_ = 1; *τ_λ_* = 1), with OD_max_ = 0.35 and normalized at the *λ*_max_ (498 nm), is plotted in Figure 1C.

#### Absolute Photosensitivity.

The absolute photosensitivity of mid-wavelength reflectance changes arising from rhodopsin bleaching can also be predicted. The molar extinction coefficient of mouse rhodopsin in solution is *ε*_max_ = 42,000 M^−1^ cm^−1^ = 42,000 cm^2^ mmol^ −1^,^29^ and taking into consideration the 1.5-fold increase in absorbance for light propagating axially in the outer segment arising from the orientation of the chromophores in the plane of the disc membrane,^20^ the molecular extinction coefficient *α_λ_*_max_ in situ is predicted to be (1.5 × 42000)/(6.023 × 10^20^) = 1.05 × 10^−16^ cm^2^ molecule^−1^. The quantum efficiency of isomerization and bleaching has been determined to have the value *γ* = 0.67,^25^ so the intrinsic photosensitivity of rhodopsin in rod discs in situ for light axially propagating along the outer segment is thus predicted to be *α_λ_*_max_*γ* = 7.0 × 10^−17^ cm^2^ = 7.0 × 10^−9^ μm^2^. The apparent or observed photosensitivity, 1/*Q_e_*, could be decreased relative to this intrinsic value by unaccounted-for transmission losses (*τ_λ_* < 1) in the prephotoreceptor ocular media, and increased if rod waveguiding concentrates light impinging on the outer segment relative to the photon density at the external limiting membrane (base of the inner segments) (*f*_wg_ > 1) (Equation 5). By combining Equation 5 and Equation 14, the absolute action spectrum *Q_e_*(*λ*) of the reflectance increase is predicted.

### Model of Fundus Spectral Reflectance of Albino and Pigmented Mice

We measured the spectral reflectance distributions of both Balb/c and C57Bl/6J mice before, during, and after bleaching exposures, and these measurements provided an opportunity to investigate a number of factors that influence reflectometry measurements. We developed a model along the lines of those previously developed by Delori and Pflibsen^11^ and van de Kraats et al.,^30,31^ and recently applied to SLO measurement of human rhodopsin.^8^ As the basic formulation of the model has been presented in these previous papers, and the modifications made for application to the mouse are modest, we present the model in Supplementary Material IV. The model was formulated to apply to both albino and pigmented mice in the dark-adapted and fully bleached states with the goal of accounting for all visible wavelength reflectance changes arising from complete rhodopsin bleaching and additional bleaching-induced broadband reflection changes observed in our studies.

## Results

### Fundus Reflection Increases Accompanying Serial Scanning With 501-nm Light

The principal paradigm used to measure fundus reflection changes is illustrated in Figure 2. Using 700-nm light, the mouse was aligned so that the image of its optic nerve head was near the center of the full field of view (full FOV, 51°), a procedure calculated to produce negligible bleaching (cf. Fig. 1). Regions lacking major blood vessels were then selected for bleaching based on retinal images acquired during the alignment (boxes in Figs. 2B, 2C outlined in unbroken red lines). Six baseline full-FOV images with 501-nm light of 1 μW were then obtained (Fig. 2A). Serial scanning of small-FOV location 1 then commenced, followed immediately by scanning of small-FOV location 2. By restricting the small field to a region one-fifth the diameter of the full field, the space-averaged energy density was increased 25-fold. At the end of 100 serial scans of both locations, a full-FOV scan showed a notable increase in 501-nm light reflected from the serially exposed regions (Fig. 2B). This increase in reflectance can be more readily appreciated by considering the difference between the 501-nm full-FOV images taken before and after the serial scanning (Fig. 2C). The areas of increased fundus reflectance were somewhat larger than the regions nominally scanned, an effect likely arising from eye displacements that accompany respiration during the ∼60-second serial scanning, and possibly from diffuse back-reflectance from the sclera of the albino mouse.

The local increases in fundus reflectance during the serial scanning were quantified by calculating from each of the stored series of images the average pixel intensity values of subregions of each scanned area (dashed boxes in Figs. 2B, 2C) of the small-FOV images; digital masks were used to exclude data from blood vessels. The pixel intensities were then plotted as a function of the scan number for both 501- and 700-nm scanning beams (Figs. 2D, 2E). The increases in reflected 501-nm light were further analyzed by fitting the geometric bleaching model of Equation 11 to the data (smooth black curves). From this analysis we obtained from each set of serial scans three parameters: *I*_0_, the initial quantity of reflected 501-nm light; Δ*I*_max_, the maximal (asymptotic) increment in reflected light; and *B*_0_, the fractional increase per scan of the small regions, which was 0.103 and 0.117, respectively, for locations 1 and 2. The percentage increases in 501-nm light, Δ*I*_max_/*I*_0_, were 100% and 90% for locations 1 and 2, respectively. In contrast, there were minimal (location 1) to undetectable (location 2) changes in 700-nm reflectance, so that adjustment for scattering (Equation 10) was inconsequential. Interpreting *B*_0_ as the fraction pigment bleached per scan, the results reveal that an individual full-FOV, 1 μW 501-nm scan should bleach ∼0.4%. Using a standard mouse rhodopsin template curve (Fig. 1B), and assuming 11% bleaching by each small-FOV 501 nm scan, the fractions of rhodopsin bleached by the 5-μW 700 nm full-FOV scans and each small-FOV 700 nm scan are estimated to be 2.2 × 10^−7^ and 1.1 × 10^−6^, respectively. These estimates confirm that alignment with 700 nm scanning (which typically takes 20–50 full-FOV scans) produced negligible bleaching. In addition, the close similarity between parameters extracted from the data of the two locations (Figs. 2D, 2E) indicates that scanning of location 1 had little effect on the data from location 2. Finally, while the 501-nm scan data illustrated in Figures 2D and 2E required no adjustment with the 700 nm data, in other instances (as illustrated below), this was not the case. In over 90 dual-band experiments the average increase in 700-nm reflectance during 100 serial scans was ∼10%, and the small adjustment for the scattering changes with Equation 10 invariably caused the adjusted 501 nm data to reach a stable asymptote slightly more quickly.

### The Photosensitivities of Albino and Pigmented Mice Are Very Similar

The normalized serial increases in reflected 501-nm light Δ*I/*Δ*I*_max_ were further examined by plotting them as a function of the cumulative energy density (Fig. 2F). The reflectance increases so plotted were then analyzed by plotting the theoretical curve generated by the complement of Equation 12, that is, *B_n_* = 1 − *p_n_* = 1 − exp[−(*n* − 1)*Q*_1_/*Q_e_*] with *Q_e._* = *Q*_1_/*B*_0_, where *Q*_1_ is the energy density of a single scan in photons μm^−2^. As expected from the good agreement of the data in Figure 2E with the geometric incrementing formula (Equation 11), the exponential rise with this directly calculated value of *Q_e_* describes the data well. Similar results obtained in 70 experiments with 7 Balb/c (albino) and 21 experiments with 5 C57Bl/6J (pigmented mice) are summarized in the Table. A remarkable feature of these data is that the values of *B*_0_ (and therefore of *Q_e_*) are very close for the albino and pigmented mice. Specifically, the mean values of log_10_ (*Q_e_*) for the two strains differ by only 0.066 units. This is not significantly different (*t*-test assuming unequal variances, *P* = 0.12). Correspondingly, the 95% confidence interval (−0.0003 to 0.131) for the difference includes zero, but notably excludes a difference between the strains greater than 0.13 log_10_ units. Moreover, even if taken as reliable, the observed difference in mean log_10_ (*Q_e_*) for the two strains, 0.066, corresponds to only 16% higher photosensitivity in albino as compared to pigmented mice. This surprisingly small difference between strains in log_10_ (*Q_e_*) suggests that light reflected from the sclera of albino Balb/c mice plays little role in causing the mid-wavelength reflectance increase.

**Table:**
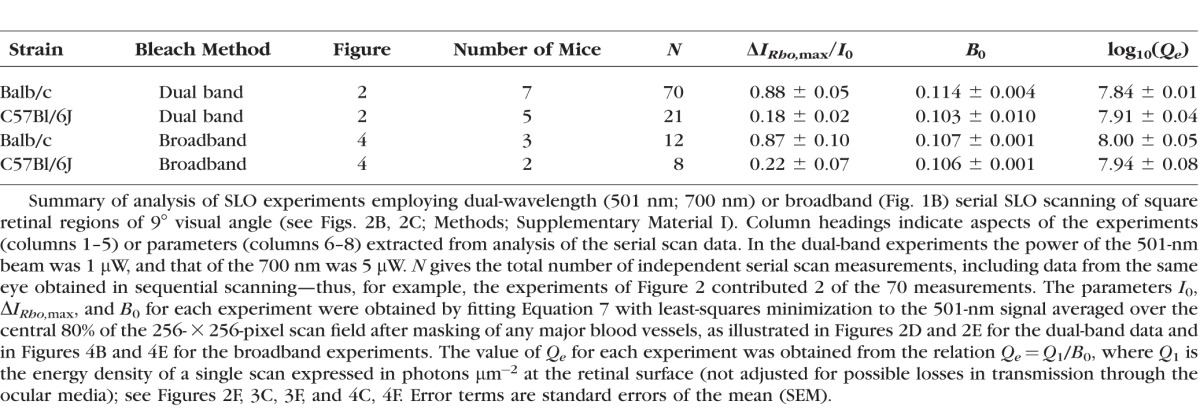
Properties of Light-Induced Increase in 501-nm Light Reflected From Mouse Fundus in SLO

### The Action Spectrum of the Mid-Wavelength Reflectance Corresponds to the Absorption Spectrum of Rhodopsin In Situ

**Figure 3:**
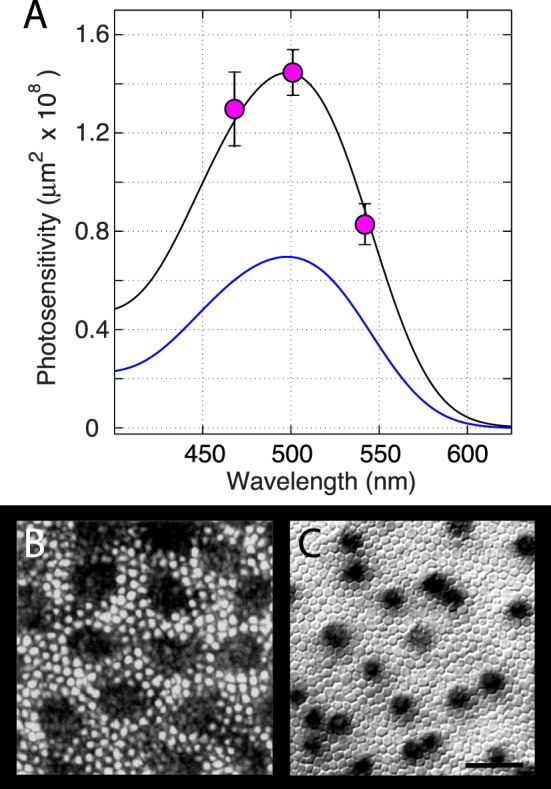
Photosensitivity of the light-stimulated increase in mid-wavelength fundus reflectance. (**A**) Plotted points give the mean ± 95% confidence intervals for 1/*Q_e_*, estimated as illustrated in Figure 2F. Experiments were performed on Balb/c mice with the paradigm of Figure 2 using bleaching stimuli of three different wavelengths. The wavelengths, numbers of mice (*N*), and experiments (*n*) were as follows: 468 nm, *N* = 4, *n* = 16; 501 nm, *N* = 7, *n* = 70; 542 nm, *N* = 4, *n* = 15. The abscissa values of the plotted points are the midpoints of the measured spectral distributions of the light transmitted by ∼10-nm bandwidth (FWHM) filters used in the experiments. The *smooth blue curve* is the prediction of the absolute photosensitivity of rhodopsin in the absence of waveguide condensation of light between inner and outer segments, namely, the absorption spectrum of mouse rhodopsin in situ (*λ*_max_ = 498 nm, OD_max_ = 0.35, *τ_λ_* = 1.0; Fig. 1C), scaled at *λ*_max_ to *α_λ_*_max_*γ* = 7.0 × 10^−9^ μm^2^. The *smooth black curve* is the same absorption spectrum scaled up by an addition factor of 2.06. The ordinate scale has been multiplied by a factor of 10^8^ for convenience of display; thus the data point at 501 nm corresponds to the value 10^−7.84^ = 1.44 × 10^−8^ as reported in row 1, column 8 of the Table. (**B**) Image of an excised, live flat-mounted piece of macaque peripheral retina homogeneously illuminated from the retinal ganglion cell side. The images of rod tips (*bright circular spots*) are much brighter than the interstices, revealing condensation of light between inner and outer segments (the *large dark regions* of the image correspond to cones, whose outer segment tips are in focus in a more anterior plane). Reprinted with permission from Packer OS, Williams DR, Bensinger DG. Photopigment transmittance imaging of the primate photoreceptor mosaic. *J Neurosci*. 1996;16:2251–2260. Copyright © 1996 Society for Neuroscience. (**C**) Image of a flat-mounted mouse retina focused at the inner segment layer. The rod inner segments are seen to tightly tile the retinal surface. The *dark spots* identify the positions of cones, labeled by diaminobenzidine reaction product. The *scale bar* is 10 μm. Reprinted with permission from Jeon CJ, Strettoi E, Masland RH. The major cell populations of the mouse retina. *J Neurosci*. 1998;18:8936–8946, Figure 1. Copyright © 1996 Society for Neuroscience.

If the increases in 501-nm reflectance are caused by light captured by rhodopsin, then the action spectrum of the increase should be proportional to the in situ absorption spectrum (Equation 14). Increases in reflectance were measureable over an almost 100-nm band centered on the *λ*_max_ (498 nm) of mouse rhodopsin in albino mice, and so we used the serial bleaching protocol (Fig. 2) to measure the photosensitivity (1/*Q_e_*) of increases in reflectance for light of several wavelengths in this band (Fig. 3A). The spectral sensitivity so measured is reasonably consistent with the predicted form of the absorption spectrum.

### The Absolute Photosensitivity of the 501-nm Reflectance Increase Is 2-Fold Higher Than Predicted in the Absence of Inner-to-Outer Segment Light Condensation

A key quantitative prediction of the hypothesis that the scanning-induced increases in 501-nm reflectance arise as a direct consequence of decreased absorption of light by rhodopsin is that the value of 1/*Q_e_* for 501-nm light should be no larger than *α*_max_*γ* = 7.0 × 10^−9^ μm^2^ (Equation 5; Methods). This prediction is illustrated in Figure 3 by the blue line: Clearly, the photosensitivity of the reflectance increases is systematically higher than predicted, despite following a rhodopsin absorption spectrum. A statistical test is provided by comparing the grand average (over both strains) of −log_10_(1/*Q_e_*) for 501-nm light with the predicted value of log_10_(*α*_max_*γ*): The grand average of −log_10_(1/*Q_e_*) was −7.86 ± 0.04 (mean ± 99% confidence interval), while log_10_(*α*_max_*γ*) = −8.15. Thus, the absolute photosensitivity 1/*Q_e_* is 2-fold (0.29 log_10_ units) greater than and very reliably (*P* < 10^−6^) different from *α*_max_*γ* calculated by assuming the light energy density guided into the rod outer segment is the same as that incident on the external limiting membrane (ELM). The simplest explanation of this 2-fold discrepancy is that the inner segments of rods, where waveguiding is initiated, condense the light by a factor of ∼2 and so increase the energy density of light propagating along the outer segment relative to that impinging on the inner segment layer. Evidence supporting the role of inner segment waveguide condensation in increasing the energy density of light propagating into the rod outer is presented in Figures 3B^32^ and 3C^33^ (taken up further in Discussion).

### Reflectance Difference Spectra During Broadband Serial Bleaching Exposures

**Figure 4:**
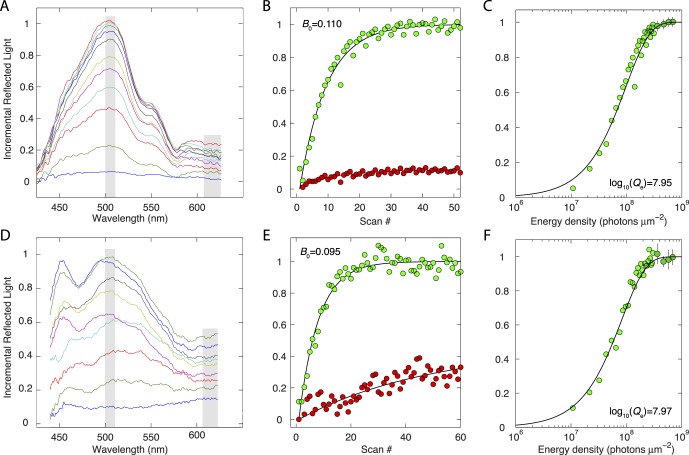
Bleaching difference reflectance spectroscopy. (**A**–**C**) Data of an albino Balb/c mouse; (**D**–**F**) data of a pigmented C57Bl/6J mouse. (**A**, **D**) Reflectance difference spectra (Equation 2) collected during serial scanning of small retinal regions as in Figure 2, but with two differences: The light source entering the pupil was a broadband spectrum (Fig. 1D); the spectrum of the light reflected from the scanned area was captured and analyzed by spectrometer 1 (Fig. 1B). The quantity plotted is the power of the light reflected from the eye with the power of the initial scan subtracted at each wavelength, and normalized overall by the saturated maximum at 500 nm that is reached after 50 to 60 scans. (For clarity, only a sample of the difference spectra is shown: For Figure 4A, the plotted spectra are from scans 3, 5, 7, 9, 11, 13, 17, 23, and 28 and the average of scans 41 to 50; for the intrinsically noisier data of the C57Bl/6 mouse of Figure 4D, the plotted spectra are averages of scans 2 and 3; 4 and 5; 6 and 7; 8 to 10; 11 to 15; 16 to 20; 21 to 30; 31 to 40; and 41 to 50. Note that the same ordinate scale applies to all parts of the figure. (**B**, **E**) The normalized difference values in the two spectral bands (mid-wavelength, 500 to 510 nm, *green symbols*; long-wavelength, 610 to 630 nm, *red symbols*; see *gray bars* in [**A**, **D**]) are plotted as a function of the scan number. The long-wavelength data (*red symbols*) were fitted with smooth curves to extract the trends over the scan series. The mid-wavelength data were adjusted as expressed in Equation 10, and the resultant data fitted with the serial bleaching model (*smooth trace through green symbols*; Equation 7; cf. Fig. 2). (**C**, **F**) Replotting of the mid-wavelength data in (**B**, **E**), respectively, as a function of the cumulative energy density to illustrate the determination of photosensitivity. The energy density in these plots is presented in rhodopsin-equivalent photons μm^−2^, calculated by integrating the measured power density spectrum (Fig. 1D) converted to photon units against the normalized mouse rhodopsin absorption spectrum template (Fig. 1C). The adjustment of the mid-wavelength reflectance data with the long-wavelength trend had negligible effect on the quality of fit and parameters extracted from the albino mouse's data, but improved the quality of the fit and caused the value of log_10_(*Q_e_*) estimated to be 0.12 log_10_ units lower than when the fitting of the pigmented mouse's data was performed without the adjustment.

We measured reflectance difference spectra (Equation 2) during serial scanning with the broadband source in pigmented and albino mice (Fig. 4). In these experiments we employed the same small-FOV scanning protocol used in the dual-band bleaching experiments, but all light reflected from the fundus was collected by the spectrometer. To facilitate analysis of the incremental changes, the data are presented as “raw” difference spectra to illustrate the relative magnitudes of the signal across the spectrum, and are normalized by the saturating level reached as the serial scanning proceeded.

In both albino (Fig. 4A) and pigmented (Fig. 4D) mice, there was a reliable increase in reflected light near 500 nm, though in both strains increases were observed in other spectral regions—notably in the long-wavelength region where neither rhodopsin nor its bleaching products would be expected to produce detectable changes in absorption. To assess whether the mid-wavelength portion of the increase in reflected light arises from rhodopsin bleaching, we applied the analyses previously used to characterize the dual-band reflectance data. The increases in reflected light in the 500- to 510-nm band in both mouse strains were well described by the geometric incrementing formula (Figs. 4B, 4E; Equation 7) with adjustment with the long-wavelength data (Equation 10), providing estimates of photosensitivity (Figs. 4C, 4F) close to those obtained in the dual-band bleaching experiments (Table).

### OCT Backscattering Data Provide a Framework for a Fundus Reflectivity Model

**Figure 5:**
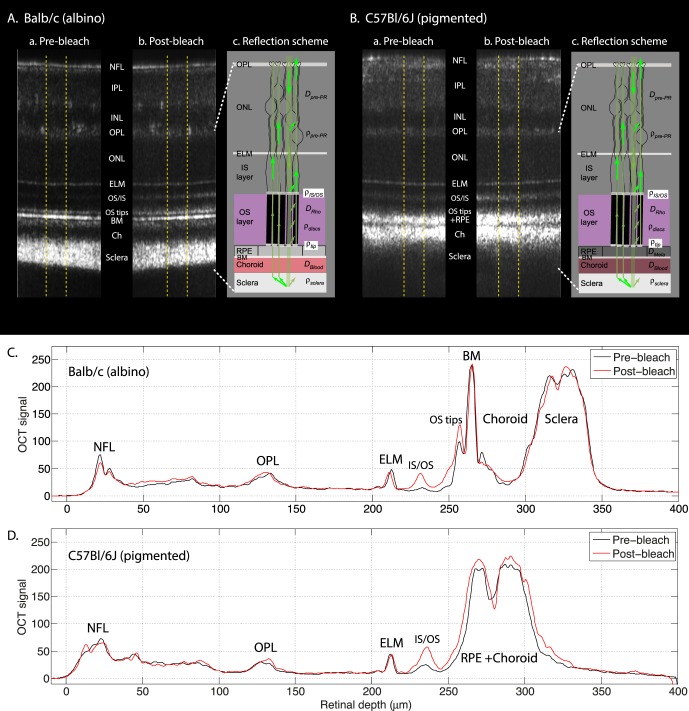
Framework for model of mouse fundus reflectivity based on OCT measurements. (**A**) OCT B-scans in the central retina of a Balb/c mouse taken (**Aa**) before and (**Ab**) after complete bleaching of rhodopsin with the protocol of Figure 2. The B-scans are presented with the grayscale lightness level linearly proportional to the scattering intensity, rather than in conventional logarithmic display mode, and the postbleach B-scan in (**Ab**) is shown in mirror image to facilitate comparison with the prebleach B-scan in (**Aa**). (**Ac**) Schematic of the paths light is assumed to take in propagating forward (*downward arrows*) and backward (*upward arrows*) through the photoreceptor layer and posterior fundus. For clarity, backward scattering by the photoreceptor discs and tip is illustrated in the rod immediately to the *right* of the one in which the forward propagating light is shown, but in reality it is confined to the same rod. More highly reflecting components (ELM), IS/OS junction, OS tips) are illustrated with *white bands*. Light transmitted through the rods does not reenter the outer segment waveguide on the return path (*arrows at left*). *Symbols* representing the parameters of the reflectance model (Supplementary Material IV, Supplementary Table S1) are given on the schematic near the retinal depth location with which they are associated. (**B**) OCT B-scans of a C57Bl/6J mouse taken (**Ba**) before and (**Bb**) after complete rhodopsin bleaching; The B-scans in (**Ba**, **Bb**) are shown in mirror image format to facilitate comparison. (**Bc**) Schematic of light propagation paths; the presence of melanin in the RPE and choroid is indicated by the *darker coloring* (compare with **Ac**). (**C**, **D**) Profile plots extracted from the OCT B-scans obtained by averaging the B-scan intensities between the *yellow dashed lines* in (**A**, **B**). The OCT B-scans and the corresponding profile plots are presented in linear, rather than in the more conventional logarithmic intensity scale display units. Retinal layers and layer boundaries are identified by conventional acronyms: neurofibrillary layer (NFL), outer plexiform layer (OPL), external limiting membrane (ELM), inner/outer segment (IS/OS), Bruch's membrane (BM).

Although the photosensitivities of the increases in 500- to 510-nm reflected light are very similar in albino and pigmented mice, there are striking differences between the difference spectra of the two strains (Fig. 4). We sought to better understand these differences by applying a model of fundus reflectivity developed by van de Kraats et al.^30^ to describe human macular reflectometry; this model was applied recently to characterize SLO measurements of human rhodopsin.^8^ We used the OCT capability of the imaging apparatus to establish a framework for modeling the layer-specific reflectance of the mouse retina (Fig. 5). Optical coherence tomography is an interferometric detection method that resolves the axial depth of retinal structures that backscatter light. Optical coherence tomography B-scans were recorded before and after 500-nm bleaching exposures in the standard protocol for both albino (Figs. 5Aa, 5Ab) and pigmented mice (Figs. 5Ba, 5Bb). The B-scans obtained before and after bleaching exposures were similar for the mouse of each strain, but bleaching-induced scattering increases in the photoreceptor layer were detected in both strains. To further characterize the effects of bleaching exposures on the OCT measurements, we extracted and plotted axial scattering profiles, OCT A-scans (Figs. 5C, 5D). These profile plots reveal bleaching-induced scattering increases at the inner segment/outer segment (IS/OS) boundary in both strains, at the OS tips in Balb/c mice, and possibly at the OS/RPE junction in C57Bl/6J mice. Not surprisingly, the regions of the eye where melanin is expressed in the pigmented mouse showed the greatest differences from the profile pattern of the albino mice. On the assumption that the distance from the RPE to the deepest layer of the sclera is approximately the same in the two strains, we interpret the differences between strains in scattering profiles in the postreceptor layers as arising from backscattering and absorption by melanin. Thus, the OCT backscattering signal from the sclera in the Balb/c profiles (Fig. 5C) is almost completely absent in the C57Bl/6J (Fig. 5D) profiles because in the choroid, the forward-propagating OCT laser beam is subject to strong attenuation by melanin absorption, as well as loss of ballistic photons from strong melanin after backscattering.

The OCT data provide a useful framework for organizing key features of the model of fundus reflectivity employed to explain the spectral effects of rhodopsin bleaching (Figs. 5A, 5Ac, 5B, 5Bc). Light propagating into the eye traverses the anterior segment, vitreous, and prephotoreceptor media in a manner independent of bleaching, with attenuation occurring in both incoming and outgoing paths. Thus, the effects of all anterior media can be lumped into two parameters, a prephotoreceptor attenuation density (*D*_prePR_) due to absorption and scattering, and a prephotoreceptor reflectance (*ρ*_prePR_) that encompasses local reflections from the neurofibrillary layer (NFL), inner and outer plexiform layers (IPL, OPL), and external limiting membrane (ELM), as well as diffuse scattering from all the prephotoreceptor layers. A large fraction of the light impinging on photoreceptor inner segments (ELM) is assumed to be coupled into the photoreceptor inner segments and guided efficiently into the outer segments (OS): Backscattering at the IS/OS junction (*ρ*_IS/OS_) from the OS tips (*ρ*_tips_) occurs, and possibly weaker backscattering by discs.^30^ During forward and backward passage through the outer segments, visible light is absorbed by rhodopsin whose density spectrum is *D_Rho_* (*λ*) (purple “OS layer” in Figs. 5Ac, 5Bc). In the albino mouse, the next major backscattering element is Bruch's membrane (BM), and light transmitted past this boundary passes on through the blood-rich choroid with total axial density *D*_blood_ (*λ*), finally to reach the highly reflective sclera (*ρ*_sclera_), where a portion of the light is backscattered and begins its return path. In the pigmented mouse, the presence of melanin in RPE apical processes near the rod outer segment tips, in RPE cells, and in the choroid adds a distinct absorbing (*D*_mela_) and backscattering element not present in albino mice. (The absence of melanin in the choriocapillaris creates a distinct dark band just below BM in the OCT profile of the pigmented mouse.)

In Supplementary Material IV we present the mathematical formulation of the fundus reflectivity model incorporating these features, and explain how it was fitted to the data. Here, we note important qualitative features incorporated into the model: (1) A large fraction of light reaching the photoreceptor layer is trapped in the inner segments and guided into the rod outer segments, which are narrower than the inner segments; (2) light is backscattered from both the base (IS/OS junction) and tips of the rod OS; (3) there is a bleaching-induced increase in backscattering by the photoreceptors' IS/OS junctions and tips; (4) light transmitted through the outer segments is backscattered by the sclera after passing twice through the postreceptor layers, where it is spectrally filtered by choroidal blood and in pigmented mice, melanin; light back-reflected from the sclera does not reenter the photoreceptor waveguides on its return path.

### The Spectra of Reflectance Increases in Albino and Pigmented Mice Can Be Explained

**Figure 6:**
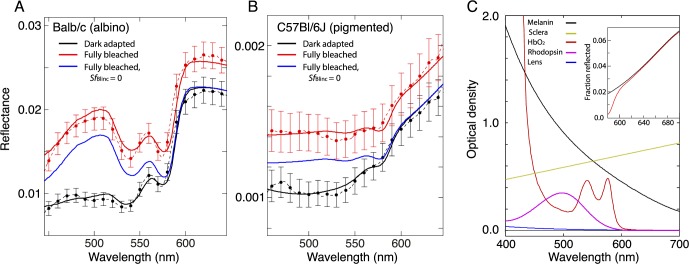
Spectral reflectance distributions of albino mice and pigmented mice described by a model of the mouse fundus. (**A**) Reflectance spectra (Equation 1) of Balb/c mice before (*black lines and points*) and after (*red lines and points*) complete bleaching of rhodopsin with the protocol of Figure 4. (Average of 12 experiments with two mice; *points and error bars* [SEM] are plotted at 10-nm intervals to illustrate variability; *dashed lines* plot the complete results obtained with the spectrometer.) The *thick, unbroken curves* plot the prediction of the best-fitting reflectance model (Supplementary Material IV; Supplementary Table S1). The *unbroken blue curve* is the prediction of the model for the full bleach case with no bleaching-induced incremental reflectance from the base and tips of rods (Supplementary Table S1, column 3 with *Sf*_Blinc_ = 0). (**B**) Reflectance spectra (Equation 1) of C57Bl/6J mice before (*black*) and after (*red*) bleaching. The data are the average of eight experiments with two mice. The *thick, unbroken curves* plot the prediction of the best-fitting reflectance model; the *blue curve* plots the prediction with no bleaching-induced incremental reflectance (Supplementary Table S1, column 4 with *Sf*_Blinc_ = 0). Note that the absolute reflectance of the C57Bl/6J mice is roughly 10% that of the Balb/c mice. (**C**) Absorption spectra (1-pass) of components of the best-fitting reflectance model presented on an optical density ordinate scale: These components combine to determine the shape of the model curves fitted to the data (cf. Supplementary Material IV for formulas). Thus, the W-shaped spectra of the albino mouse in (**A**) arise from absorption by oxygenated hemoglobin (HbO_2_, *dark red curve* in [**C**]), while the positively sloped linear segment of the long-wavelength region of the reflectance spectra of the pigmented mouse in (**B**) arises from the product of the scleral reflectance spectrum multiplied by the 2-pass density of RPE and choroidal melanin. To illustrate this latter point, *ρ*_sclera_(*λ*)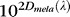 (*black curve*) and *ρ*_sclera_(*λ*)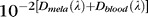 (*red curve*) are plotted in the *inset* in the *upper right* of (**C**) in linear ordinate units on the common wavelength scale used.

We fitted the fundus reflectance model to the average absolute reflectance spectra obtained before and after a full bleaching exposure (Fig. 6). This presentation of the spectral reflectance data contrasts with that in Figure 4 in three ways: (1) The data are presented in absolute units, so that the absolutely scaled light reflected from the eye has been divided wavelength by wavelength with the absolute power distribution of light entering the eye (Equation 1); (2) only data of the dark-adapted and fully bleached states are presented; (3) the results have been averaged over a series of experiments with each strain of mice.

Good descriptions of the data of both Balb/c and C57Bl/6J mice were obtained; the parameters of best fit are presented in Supplementary Table S1 of the Supplementary Material. The model accounted for the following highly distinctive features of the data as follows. (1) The “W-shaped” dips in the spectra of albino mice between 520 and 600 nm arise from absorption by oxygenated hemoglobin in the choroid of albino mice (Fig. 6A); (2) the linear increases in reflectance with wavelengths above ∼590 nm in pigmented mice arise from a combination of the decreasing melanin absorbance at long wavelengths and increasing scleral reflectance (Figs. 6B, 6C); (3) the increases in reflectance above 600 nm (where rhodopsin absorption is negligible) in both strains can be explained by light-induced incremental reflectances at the IS/OS junctions and tips of rods; (4) increases in reflectivity around 500 nm with bleaching exposures in both strains can be explained by a combination of loss of rhodopsin absorption and spectrally flat increases in reflectivity at the IS/OS junction and tips of rods. We now briefly expand the explanations of these phenomena.

#### W-Shaped Spectra in Balb/c Mice.

The distinctive W-shaped fingerprint of oxygenated hemoglobin (HbO_2_) absorption in the Balb/c reflectance spectra is unmistakable (Fig. 6C). The fitted HbO_2_ absorption spectrum corresponds to an average concentration of 2.0 mM in the measured ∼45-μm-thick choroidal layer (cf. Fig. 5C).

#### Long-Wavelength Reflectance Increases Linearly With Wavelength in C57Bl/6J Mice.

The distinctive linear pattern of long-wavelength reflectance of the pigmented mice arises from the product of the spectral dependence of the scleral reflectivity coefficient and that of melanin absorption. Thus, the product of the scleral reflectivity coefficient and melanin absorption coefficient produces a linear increase in long-wavelength reflectivity (Fig. 6C, inset). This conclusion appears to be at odds with the OCT data of pigmented mice (Fig. 5D), which suggest that little light penetrates to the sclera. A possible resolution would be that melanin backscattering in pigmented mice plays a spectral shaping role similar to that of the sclera in albino mice.

#### Bleaching-Induced Increases (“Blinc”) in Long-Wavelength Reflectance.

As expected from the absorption spectrum of rhodopsin in mouse rod (Fig. 1C), we found that the increases in long-wavelength reflectance upon bleaching could not be explained by loss of absorption due to bleaching, and found that instead the increases could be explained by a 35% light-induced, spectrally neutral bleaching-induced increment in reflectance at the IS/OS junctions and tips of rods (Supplementary Table S1, *Sf*_Blinc_). Motivation for including this “Blinc” feature in the model was provided by OCT data (Figs. 5C, 5D). When the Blinc factor was deleted (*Sf*_Blinc_ = 0), the model failed systematically across the entire spectrum for both strains (blue curves in Figs. 6A, 6B). For simplicity (Occam's razor) the Blinc factor was assumed to be spectrally neutral, but a more complex spectral dependency (such as would arise from Raleigh scattering) is possible and remains to be explored.

#### Dominant Contribution of Rhodopsin Bleaching to the Increase in Reflectance in the Spectral Neighborhood of 500 nm.

**Figure 7:**
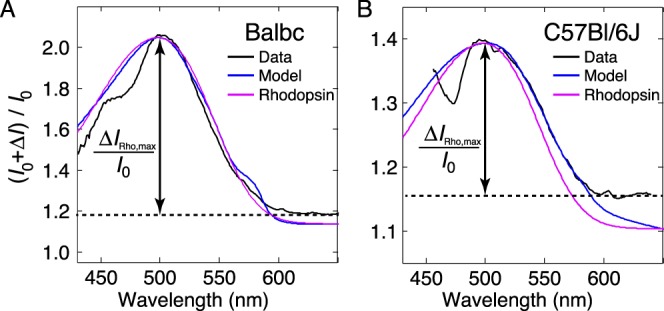
Mid-wavelength increases in reflectance spectra of Balb/c and C57Bl/6J mice peak are centered at 500 nm. (**A**) The ratio of the reflectance spectra of Balb/c mice taken after (Fig. 6A, *red curve*) to that taken before bleaching (Fig. 6A, *black curve*) is plotted as the unbroken noisy *black trace*. The *blue curve* is the ratio of the corresponding curves in Figure 6A generated with the model. The *magenta curve* is the absorption spectrum of mouse rhodopsin in a rod with an axial density (OD_max_) of 0.35 at the *λ*_max_ (498 nm). (**B**) Ratio of reflectance spectra of C57Bl/6J mice taken after (Fig. 6B, *red curve*) to that taken before bleaching (Fig. 6B, *black curve*). The *blue curve* plots the corresponding ratio of the model curves from Figure 6B, and the *magenta* the same rhodopsin absorption spectrum as in (**A**).

The reflectance increases in the spectral band around the *λ*_max_ (498 nm) of mouse rhodopsin in the two strains of mice were accounted for by a combination of two factors: loss of rhodopsin absorption, and the aforementioned spectrally neutral reflectivity increases at the rod IS/OS junctions and tips. The same rhodopsin absorption spectrum and the incremental reflectance factor (Supplementary Table S1, *Sf*_Blinc_) parameter values were obtained in modeling the reflectance data of both strains of mice, despite their highly distinctive overall reflectance distributions. To further elucidate the dominant contribution of rhodopsin bleaching to the mid-wavelength reflectance increases, we plotted (*I*_0_ + Δ*I*)/*I*_0_, that is, the ratio of the after- to before-bleach reflectance spectra, along with the model curves and the rhodopsin absorption template (Fig. 7). The rhodopsin spectrum was scaled to match the amplitude and extent of the model curve, but no other scaling was used. These results are consistent with the hypothesis that the dominant fraction of the mid-wavelength reflectance increase arises from light that makes only one pass through the rods.

#### Additional Spectral Feature.

Unadjusted for the spectral distribution of the broadband source spectrum entering the eye, the raw bleaching difference reflectance spectra of the pigmented mouse exhibit a dip centered around 460 to 470 nm, which appears to increase in magnitude with successive scans (Fig. 4D). While not obviously present in the raw reflectance data of albino mice (Fig. 4A), a similar feature does appear in the ratios of the average spectral reflectances before and after bleaching of both mouse strains (Fig. 7). We suggest that this feature could arise from absorption by metarhodopsin III (MetaIII). The latter is a spectroscopically identified (*λ*_max_ ∼465 nm) rhodopsin bleaching product in which the isomerized all-*trans* chromophore remains bound to opsin, pending chromophore hydrolysis.^9^ Approximately 30% of rhodopsin bleached is transiently converted to MetaIII,^34^ and in humans the concentration of MetaIII reaches its maximum around 1 minute after the bleaching exposure.^9^ Unfortunately, the short-wavelength region of the spectrum of the broadband source has relatively low power (Fig. 1D, blue, black curves), precluding a quantitative analysis of this component based on the present data, and it remains a target for future investigation.

### Limitations of the Spectral Reflectance Model: Parameter Tradeoffs and Omissions

While able to generate a reasonable account of the main features of the fundus reflectance spectra of dark-adapted and fully bleached albino and pigmented mice (Figs. 6, 7), the reflectance model has notable limitations. First, the mouse retina has a number of relevant features distinct from those of the human retina, for which the model was originally formulated.^8,30^ Thus, for example, the depth location and relative strength of backscatter bands in OCT profile plots differ between humans and mice, and the density and depth distribution of melanin in the posterior pole of pigmented mice appear to be substantially greater than in the average human eye. Second, there are unavoidable, inherent tradeoffs between some parameters in fitting the reflectance data, in particular, between several spectrally neutral absorbance and reflectance parameters (*D*_prePR_, *ρ*_prePR_, *D*_deep_, *Sf*_confocal_*, ρ*_IS/OS_, and *ρ*_tips_), and as a consequence the parameters of best fit are not uniquely determined. Third, the inability of the current model to provide a good description of the reflectance data of both albino and pigmented mice with similarly valued parameters for all model components anterior to the RPE (the first layer of melanin pigmentation) suggests that at least as applied to pigmented mice, the model may have a serious defect. From consideration of the OCT data (e.g., Figs. 5C, 5D) we suspect that this defect arises from neglect by the model of backscattering by melanin in the RPE and choroid, and its forcing of all “deep reflected” light to arise from the sclera. Thus, comparison of the OCT data of albino and pigmented mice indicates that little 860-nm light penetrates past the melanin-containing RPE and choroidal layers to the sclera. In addition, there could also be a mutually nonexclusive defect in the formulation of the preretinal ocular media scattering loss density (*D*_prePR_) and reflectance (*ρ*_prePR_) parameters, which have yet to be fully characterized in both pigmented and albino mice. In presenting these caveats, we nonetheless emphasize that the four main conclusions drawn from the application of the spectral reflectance modeling (see previous section) are robust against parameter tradeoffs, and that in particular the model components incorporating spectrally dependent factors—rhodopsin, oxygenated hemoglobin, and melanin shaping of the long-wave portion of the reflectance spectra of pigmented mice (Fig. 6C; Supplementary Table S1)—are well determined. Finally, in both albino and pigmented mice the total reflectance increase caused by complete rhodopsin bleaching could not be described by the model without incorporating a bleaching-induced increase in reflectance at the base and tips of the outer segment, in addition to mere loss of rhodopsin absorption.

## Discussion

We have presented multiple lines of evidence that the bleaching of rhodopsin in vivo in albino and pigmented mice can be measured with a custom scanning laser ophthalmoscope (SLO). First, the absolute photosensitivity of the increase in reflected 501-nm light is close to that expected for rhodopsin in situ (Methods; Fig. 2; Table). Second, the action spectrum of mid-wavelength reflectance increases is well described by the absorption spectrum of rhodopsin in situ (Fig. 3). Third, the photosensitivity of mid-wavelength reflectance increases induced by broadband light converted into rhodopsin-equivalent photons is close to that obtained with monochromatic light (Fig. 4; Table). Fourth, ratiometric spectroscopy of the spectral reflectance distributions shows that bleaching of rhodopsin is the predominant contributor to incremental reflectance in both strains (Figs. 6, 7). While thus establishing that rhodopsin bleaching in living mice can be quantified with an SLO, our results also reveal several features of bleaching-induced changes in mouse fundus reflectance that call for further interpretation and discussion.

### Light Reflected From the Posterior Fundus Does Not Readily Enter the Rod Outer Segment

A remarkable feature of our data is that the photosensitivity of rhodopsin bleaching is only 16% higher in albino (Balb/c) and than in pigmented (C57BL/6J) mice (Table). This near equivalence implies that primarily light entering the rod outer segment waveguide from the inner segment side contributes to rhodopsin bleaching during SLO scanning. Thus, although reflections from the postreceptor layers are much greater from albino as compared to pigmented mice (Figs. 5, 6), a surprisingly negligible amount of this deep-reflected light appears to be capturable by rhodopsin in albino mouse rods. Such a “one-way” waveguiding feature has been previously inferred to apply to human cone photoreceptors and has been incorporated into a successful model of human cone pigment retinal reflectometry,^30^ but this principle has not generally been thought to apply to rods.

### Resolution of the 2-Fold Discrepancy From Prediction of Rhodopsin Photosensitivity

The measured photosensitivity of rhodopsin bleaching (1/*Q_e_*) in both albino and pigmented mice (Fig. 3, Table) is approximately 2-fold greater than predicted by the photosensitivity of rhodopsin in vitro, combined with the effect of the in situ dichroism (Methods), and the assumption that the spatial density of photons entering the rod outer segment waveguide is equal to that impinging on the inner retina. A similarly elevated photosensitivity of human rhodopsin^4,8^ and even more highly elevated photosensitivity of human cone pigment bleaching have long been noted. These higher than predicted photosensitivities cannot arise from unaccounted-for transmission losses in the ocular media or from the presence of the pigments in density in the outer segments, as both of these factors would act to decrease, not increase, the apparent photosensitivity. The likely explanation for the elevated photosensitivity is light condensation between rod inner segments and outer segments, as now discussed.

Initiation of waveguide capture of light by inner segments is well established for cones, and is associated with a tuning of the acceptance angle, the Stiles-Crawford (SCI) effect.^35,36^ For example, the relatively large inner segments of turtle cone clearly “focus” or guide light onto the much narrower outer segments, and produce a strong SCI effect.^37^ Although it is often stated otherwise, human rods do exhibit an SCI effect, albeit one weaker than that of cones.^38^ Thus, a supposed absence of a rod SCI effect is not a compelling argument against the hypothesis that waveguiding in rods, as in cones, begins in the inner segments. Moreover, when light from homogeneously illuminated flat-mounted retinas is imaged as it emerges from rod outer segment tips, the nonuniform spatial pattern very clearly demonstrates condensation by the inner segments (e.g., Fig. 3B^32^). In addition, images of flat-mounted retinas focused at the photoreceptor inner segment layer invariably show the inner segments to tightly tile the retinal surface (Fig. 3C^33^; see also www.cis.uab/edu/sloan/curcio/HOUSTON [in the public domain]). Tight surface tiling at the inner segment layer is also observed in adaptive optics imaging of human photoreceptor mosaics, including ones in the retinal periphery where rods are prevalent.^39^ The adult mouse retina has a total surface area of ∼18 mm^2^,^23,33^ an area approximately twice that of the total cross section of the base of all the rod outer segments. (Cones comprise only 3% of mouse photoreceptors, and while their inner segments are larger than those of rods, they certainly contribute well less than 10% of the total inner segment cross-sectional area.) Thus, given that the total number of rods in the adult C57Bl/6 mouse is 6.4 × 10^6^,^33^ and that each outer segment has a diameter of 1.4 μm^24^ and a corresponding cross-sectional area of 1.54 μm^2^, the total rod outer segment cross-sectional area is 9.8 × 10^6^ μm^2^ or 9.8 mm^2^, approximately half the total retinal area. Hence, if most of the light incident on the mouse inner retinal surface is captured and guided by rod inner segments to the outer segments, the apparent discrepancy in photosensitivity of rhodopsin can be considered resolved: Rod waveguide condensation of light causes the photon density at the outer segment layer to be increased by ∼2-fold (*f*_wg_) relative to that at the inner segment, where guiding begins.

### Mouse Fundus Reflectivity in the Dark-Adapted and Bleached States

Several distinctive phenomena were observed in measurements of the fundus reflectivity of Balb/c and C57Bl/6J mice, including a W-shaped reflectance spectrum in albino mice, a linearly increasing long-wavelength reflectivity in the pigmented mice, and light-induced increases in longwave reflectivity in both strains (Fig. 6). To gain insight into these phenomena we developed and applied a fundus reflectance model along the lines of that developed by van de Kraats et al.^30^ for human cones (Fig. 5; Supplementary Material IV). The fitted model provided a good account of the reflectance data of both strains (Fig. 6), with reasonably interpretable parameter values (Supplementary Table S1). For example, fitting of the model to the reflectance spectra of Balb/c mice estimated the average concentration of HbO_2_ in the 45-μm-thick choroidal layer to be 2 mM (cf. Fig. 5C). The concentration of HbO_2_ in oxygen-saturated mouse blood can be calculated from published data about mouse blood volume (1.1 mL for a 20-g mouse), hematocrit (0.5) red blood cell volume (RBC; 56 μm^3^), and hemoglobin (3.9 μmol/RBC) to be 3.6 mM. Thus, the fractional hemoglobin volume of the Balb/c mouse choroid is estimated to be 2/3.6 × 100 = 56%. For the C57Bl/6J mice the estimated concentration of HbO_2_ was lower than for Balb/c, but this can be interpreted to mean that due to melanin absorption the effective thickness of the blood layer through which light back-reflected from postphotoreceptor structures travels is smaller (compare Figs. 5C, 5D).

### Near-Infrared Light Can Serve to Adjust for Broadband Scattering Changes

Long-wavelength backscattering changes cannot arise directly from diminished absorption arising from bleaching of rhodopsin, which, along with its photoproducts, absorbs negligibly at wavelengths longer than approximately 600 nm. Such scattering changes could affect and potentially distort measurements by fundus reflectometry of visual pigment properties such as photosensitivity, if parallel changes occur in the visible spectrum.^40^ Historically, most human retinal reflectometry has simultaneously measured mid- and long-wavelength reflected light and used various strategies to correct for distortions (e.g., Refs. 4, 8, 27). In this study of mice we observed bleaching-induced increases in deep red reflectance with both SLO (Figs. 4, 6) and OCT measurements (Fig. 5). Analysis with the reflection model suggests that while detectable with NIR light, the mechanisms underlying these changes also increase reflectance in the visible portion of the spectrum, and can largely be attributed to increased back-reflectance at the rod IS/OS junction, the OS tips, and perhaps throughout the outer segment. Thus, if the bleaching-induced component of incremental reflectance of the model is eliminated (*Sf*_Blinc_ = 0), the model cannot account for the total reflectance increases in both the mid- and long-wavelength regions (Figs. 6A, 6B, blue curves). Given the success achieved by incorporating photoreceptor-specific reflectance increases, we think that it remains reasonable in fundus reflectometry work to correct mid-wavelength reflectance measurements with simultaneously collected deep red or NIR reflectance measurements (e.g., Figs. 4E, 4F) on the assumption that the incremental reflectance is spectrally flat (Equations 6, 7).

### Mechanism of Light-Induced Fundus Reflectance Changes Not Arising From Decreased Rhodopsin Absorption

Visible light-induced NIR retinal scattering changes were first reported many years ago,^41^ and have been investigated with IR-OCT (reviewed in Ref. 42; see also Ref. 43). Our OCT results reveal that photoactivation of rhodopsin triggers increases in NIR backscattering at the IS/OS junction and at the OS tips (Figs. 5C, 5D), which as mentioned above cannot arise from decreased rhodopsin absorption. We found that incorporating photoreceptor-specific reflectance increases at the base and tips of the outer segments into the reflectance model was necessary and sufficient (Figs. 6A, 6B) for the model to describe the broadband reflectance data, which extends to long wavelengths where rhodopsin absorption is negligible. The cellular and molecular mechanisms underlying these photoreceptor reflectance increases remain to be determined and are a focus of ongoing investigation. It also remains to be determined whether the backscattering changes in human cones^44–46^ measured with AO-SLO and AO-OCT arise from mechanisms similar to those operative in mouse rods.

## Supplementary Material

Supplement 1
