# Supplementary material for: The Photosensitivity of Rhodopsin Bleaching and Light-Induced Increases of Fundus Reflectance in Mice Measured In Vivo With Scanning Laser Ophthalmoscopy
Source: Invest Ophthalmol Vis Sci. 2016 Jul 12;57(8):3650–64. doi: 10.1167/iovs.16-19393 (PMC4959838; doi:10.1167/iovs.16-19393)
Supplement: Supplement 1 [file i1552-5783-57-8-3650-s01.pdf]

I. Conversion of SLO scanning angles to retinal distances*

A goal of this investigation was the determination of the photosensitivity of rhodopsin bleaching in the living mouse eye. As the SLO was used to deliver bleaching stimuli (**Fig. 2**), accurate determination of the retinal area to which the stimuli were delivered was critical. The optical parameters of the adult mouse eye have been well characterized[1-3] and a simple conversion from scan angle in radians to linear distance on the retina with using a standard posterior nodal distance[1-3] (PND) of 1.95 mm at first seemed appropriate. We were concerned, however, that the use of a contact lens and gel might increase the PND. We thus undertook an experiment in which we first used the SLO to image a mouse retina whose blood vessels had been rendered fluorescent and then after sacrificing the mouse imaged the flat-mounted retina in a confocal microscope (**Fig. S1**). This experiment revealed the calibration between scan angle and retinal distance to be 43 µm deg^{-1} (**Fig. S1**) and we have used this to calculate the scan area of the standard small-FOV in the bleaching experiments to be 387 µm × 387 µm (**Fig. 2**).

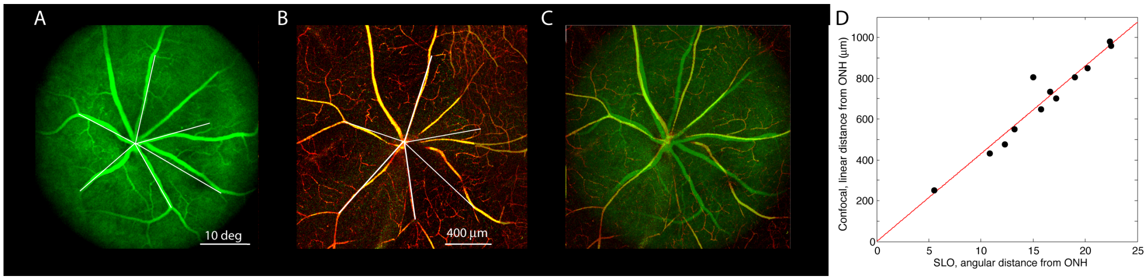

Fig. S1. Calibration of the scan field. **A.** Full-FOV SLO angiographic image of the retina of a mouse injected by tail vein with fluorescein. **B.** Image of the flat-mounted eye of the same mouse taken with a Nikon A1 confocal microscope (4X); the images in B has been arranged so as to best correspond to that in A, as illustrated in **C,** which presents a superposition of the two. The Nikon A1 image scale was calibrated with a 10 µm reticle grid and found to be accurate to within 1%. **D.** A number of retinal vessel branch-points could be unequivocally identified in both images A and B (see white lines superimposed on the images for examples), and the distances of the branch points from the center of the optic nerve head measured independently in the two images in their respective units. These measurements were plotted against one another and fitted with a straight line forced to go through the origin (red line). The slope was found to be 43.1 µm deg^{-1}.

* In the supplementary material, figures in the manuscript text are referred to in the same manner as in the text. Figures and equations that appear only in the supplementary material are given labels that begin with "S": e.g., Fig S1, Eq S1.

II. Absolute calibration of the broadband laser light source at the pupil

In a number of the experiments presented we employed a broadband supercontinuum light source (Fianium, SC-400) in order to measure mouse fundus spectral reflectance distributions over a wide spectral range before, during and after bleaching (**Figs. 4, 6**). To determine the absolute reflectances (Eq 1) in these experiments it was necessary to measure $P_{in}(\lambda)$, the absolute spectral power density (watts nm^{-1}) of the source at the pupil plane (**Fig. 1D**). To extract a spectrum proportional to $P_{in}(\lambda)$ we created a model eye with a Fluorilon 99W reflecting disc (Avian Tech, Gainesville, FL); the latter acts as a Lambertian reflector with an extremely (< 0.5%) flat reflectance spectrum. We used manufacturer specifications for the SLO optical elements to correct for losses in transmission from the disc to the spectrometer, but in fact found the predicted losses to be very small (**Fig. 1D**). The absolute spectral density was then determined by equating the power measured with the Thorlabs power meter with the numerically integrated spectrum measured by the spectrometer from the light reflected through the optical system from the model eye, as now described.

First, consider a monochromatic laser stimulus of wavelength λ and power 1μW, and identify the spectral power density of this stimulus as $\boldsymbol{P}_{1\mu W}(\lambda)$. The Thorlabs S120C power meter uses a silicon photodiode operating in photovoltaic mode: it first converts the current generated in response to light into voltage, and then, internally converts the voltage into a power reading. The current that $\boldsymbol{P}_{1\mu W}(\lambda)$ generates in the power meter is $P_{1\mu W}(\lambda)S_{PM}(\lambda)$ (amps), where $S_{PM}(\lambda)$ is the spectral sensitivity of the power meter expressed in amps/watt. The internal voltage generated is $K\,\boldsymbol{P}_{1\mu W}(\lambda)$ $S_{PM}(\lambda)$, where K must have the unit ohms. When using the power meter the user must select a wavelength (in this case λ itself), and the internal (digital) circuitry of the meter then generates an appropriate scale factor $C_{PM}(\lambda)$ such that the readout is 1 μW. The internal scale factor must satisfy

$$P_{PM}(\lambda) = C_{PM}(\lambda) K [S_{PM}(\lambda) P_{1\mu W}(\lambda)] \qquad \text{Eq S1}$$

because $P_{PM}(\lambda)$ and $\boldsymbol{P}_{1\mu W}(\lambda)$ are both equal to 1 μW. It follows that the power meter scale factor $C_{PM}(\lambda)$ is given by

$$C_{PM}(\lambda) = 1 / K\, S_{PM}(\lambda) \qquad \text{Eq S2}$$

i.e., the scale constant $C_{PM}(\lambda)$ is inversely proportional to $S_{\mathrm{PM}}(\lambda)$. These relations are not surprising: they simply recapitulate the procedure by which the photodiode-based power meter converts the current generated by a monochromatic stimulus into a power reading.

Next consider an experiment with a *broadband* source having unknown power spectral density $P_{in}(\lambda)$ (watts/nm) at the pupil plane. With the Ocean Optics spectrometer we measure the light from this source reflected through the imaging system from the model eye. We used manufacturer specifications for the SLO optical elements to correct for spectral distortions in transmission from the model eye to the spectrometer, even though the distortion was very small – **Fig. 1D**). These measurements and corrections yield a measured spectral power density $P_{ME}(\lambda)$ from the model eye that is proportional to the light spectral power density entering the eye: i.e., $P_{in}(\lambda) = C_{SF}\, P_{ME}(\lambda)$, where C_{SF} is a scale constant to be determined. In each experiment we took a reading with the Thorlabs power meter from the broadband distribution with the calibration wavelength set (arbitrarily) to 580 nm, so that the power meter used the internal scale factor C_{PM} (580 nm) for its conversion. The measured power reading P_{PM} (watts) must then satisfy the relation

$$\begin{aligned} P_{PM} &= \int C_{PM}(580nm)\, K[S_{PM}(\lambda) C_{SF}\, P_{ME}(\lambda)]\, d\lambda \\ &= C_{SF} \int \frac{K[S_{PM}(\lambda)\, P_{ME}(\lambda)]}{K\, S_{PM}(580nm)} d\lambda \end{aligned} \qquad \text{Eq S3}$$

where in the second line of Eq S3 substitution from Eq S2 was made. It follows that the scaling factor C_{SF} is obtained as

$$C_{SF} = \frac{P_{PM}}{\int \frac{S_{PM}(\lambda)}{S_{PM}(580nm)} P_{ME}(\lambda)\, d\lambda} \qquad \text{Eq S4}$$

Thus, the desired absolute spectral power density $P_{in}(\lambda)$ of light entering the eye was obtained from the measured (and transmission-corrected) spectrum $P_{ME}(\lambda)$ of the model eye and the derived scale factor as $P_{in}(\lambda) = C_{SF}\, P_{ME}(\lambda)$. A useful feature of Eq S4 is that the ohmic constant K in Eq S3 is eliminated, and only the relative spectral sensitivity of the power meter is needed because S_{PM} appears as a ratio. In the determination of C_{SF} the denominator of Eq S4 was obtained by interpolating the measured model eye

spectrum $P_{ME}(\lambda)$ and the Thorlab power meter sensitivity spectrum $S_{PM}(\lambda)$ on a 1 nm grid and performing trapezoidal numerical integration with a Matlab™ script.

The units of the broadband spectrum $P_{in}(\lambda)$ are W/nm. We converted the latter into rhodopsin-equivalent (λ_{max} = 498 nm) photons by converting $P_{in}(\lambda)$ into photon flux units and integrating against the absorption spectrum of mouse rhodopsin, $Rho(\lambda)$:

$$\Phi_{Rho-eq} = \int P_{in}(\lambda)(\lambda / hc) Rho(\lambda)\, d\lambda \qquad \text{Eq S5}$$

where "*h*" is Planck's constant and "*c*" is the speed of light, and $Rho(\lambda)$ is the absorption spectrum derived from Lamb's[4] photopigment extinction spectrum template with λ_{max} = 498 nm as modified by Govardovskii[5] to accommodate the pigment β-band, and converted to an absorption spectrum with axial density OD_{max} = 0.35 at the λ_{max} (cf. **Fig. 1D**, red curve). A test of the effectiveness of the absolute calibration and the conversion to rhodopsin-equivalent quanta is provided by comparison of the photosensitivities of rhodopsin bleaching obtained with monochromatic 501 nm light and with the broadband source (Table 1).

III. Correction of the photosensitivity for pigment axial density

In this work we characterized serial bleaching scan data (**Figs. 2, 4**) with an exponential function of the integrated energy density (Eq 4), and used this latter formula to estimate the photosensitivity ($1/Q_e$) of rhodopsin *in vivo* (Table 1; Fig. 3). As discussed in METHODS, this formula implicitly assumes that pigment is present in "low density" in rods, i.e., that the optical density for light propagating axially in the outer segment is less than about 0.2. However, abundant microspectrophotometric literature[6-8] and other arguments[9] have established that the specific axial density of rhodopsin in rods is 0.014 to 0.018 OD/µm, so an average length mouse rod outer segment of 22 µm (ref [10]) will have OD_{max} = 0.31 to 0.40, in conflict with the "low density" assumption. Thus, it seems problematic to employ the exponential decay formula (Eqs 4, 12) to quantify bleaching dependence on energy density.

In METHODS, we present the rate equation (Eq 3) appropriate for bleaching when the pigment is present "in density". As noted, the solutions to Eq 3 for $OD_\lambda > 0.2$ are not separable in *p*, the fraction pigment present after the bleaching exposure, but can be obtained by numerical analysis so that *p*, the fraction pigment present, can be plotted

as a function as a function of the energy density $Q = I_{inc}\, t$. In **Fig. S2** we present the predicted dependence of p on Q for two cases, $OD_{\max}$ = 0.35 (the value used throughout the manuscript) and $OD_{\max} \rightarrow 0$, or "low density". The form of the solution of Eq 3 for $OD_{\max}$ = 0.35 and the low density case are very similar, as seen by shifting the curve for $OD_{\max}$ = 0.35 to the left by 0.107 $\log_{10}$ units (**Fig. S2**, red curve), i.e., a factor of 1.28 on the intensity axis. From the similar form of the red and black curves,

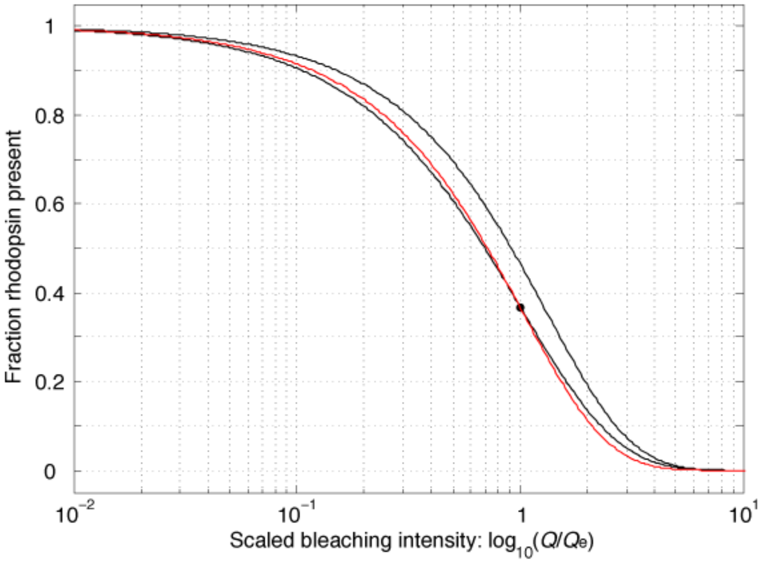

Figure S2. Effect of rhodopsin axial density on the bleaching function. Black traces plot solutions of Eq 3, i.e., the fraction of rhodopsin remaining after exposure of the retina to a bleaching stimulus whose energy density is expressed in units of $Q_e = 1/(\alpha\gamma f_{wg})$: the black curve at right is the solution for $OD_{\max}$=0.35, while that at left is for $OD_{\max} \rightarrow 0$. The red curve is the curve for $OD_{\max}$=0.35 shifted leftward by 0.107 $\log_{10}$ units.

it is clear that both forms would fit the data well (cf. **Fig. 1D, E; Fig. 4B, E**), and this justifies using the simpler, exponential decay form for least-squares fitting to the data. However, a consequence of using the exponential form is that the value of Q_e will be overestimated by the shift factor that brings the curve corresponding to the true value of $OD_{\max}$ into best correspondence with the exponential ("low density") case. This follows, because the original black curves are plotted with an abscissa scaled by Q_e: for $OD_{\max}$ = 0.35 the abscissa value $Q/Q_e = 1$ corresponds *not* to $p = 1/e = 0.37$, but rather to p = 0.457. Thus, if the true value of $OD_{\max}$ is 0.35, the values of $\log_{10}(Q_e)$ in Table 1 should all be decreased by 0.107 $\log_{10}$ units. We have not made this correction, however, for the following reasons. First, the exponential form provides a readily used parametric description of bleaching data. Second, comparison between photosensitivity investigations from different labs may be facilitated by use of the simpler, exponential decay formula. Third, at least two other parameters -- f_{wg} and $OD_{\max}$ -- need to be known to use Q_e to estimate $\alpha_{\max}\gamma$, the intrinsic photosensitivity. While we provide an argument

for the estimate $f_{wg} \approx 2$ (Discussion) and also show that the value $OD_{max} = 0.35$ is consistent with our reflectance data (**Fig. 6**) and microspectrophotometry, these values may not definitive, and certainly may be different in different species (f_{wg}) and in different mouse strains, different parts of the retina, and different rearing conditions, which are know to alter the outer segment length and thus OD_{max}. Finally, we note when serial bleaching data (**Fig. 2**) are fitted with the "univariant" exponential formula (Eq 4, 12), the resultant action spectrum $1/Q_e(\lambda)$ will be proportional the absorption spectrum (Eq 14). In contrast, if bleaching data for a series of different wavelengths simultaneously fitted with solutions to Eq 3 with the "true" value of OD_{max} the resultant action spectrum $1/Q_e(\lambda)$ should be proportional the extinction (low density) spectrum of rhodopsin. This follows because solutions to Eq 3 automatically adjust for the "top flattening" effect of optical density on the absorption spectrum.

IV. Reflectance model of albino and pigmented mouse eyes

Albino and pigmented mice have qualitatively different fundus spectral reflectance distributions, which also are altered in distinctive ways by bleaching (**Fig. 6**). To explain these differences we developed a model along the lines of those previously developed by Delori and Pflibsen[11] and van de Kraats *et al.*[12, 13] and recently applied to SLO measurement of human rhodopsin[14]. Schematics of the light flow paths identifying some of the major reflective and absorptive components of the fundus are illustrated in **Fig. 5Ac** and **Fig. 5Bc**. Here we provide the mathematical formulation of the model and describe the procedure used in fitting it to the spectral reflectance data of the two mouse strains.

Components and parameters of the mouse eye reflectance model.

Components of the model are described in the subsequent paragraphs. The parameter of the model are described in the context of presenting **Fig. 5** of the manuscript, are listed in Table S1 and their role in equations described below.

Absorbing components. The model formally included four spectrally varying absorbing components: the lens, melanin in the RPE and sclera (C57Bl/6J only), oxygenated hemoglobin (HbO_2) in the choroid, and rhodopsin in the outer segment layer. These components are associated with wavelength-dependent optical density spectra

$D_{lens}(\lambda)$, $D_{mela}(\lambda)$, $D_{blood}(\lambda)$ and $D_{Rho}(\lambda)$. A lens absorption spectrum was extracted from published transmission spectra of the rat lens[15] (blue curve in Fig. 6C). These data strongly confirm that the rodent lens is highly transmissive in the near UV (and throughout the visible spectrum), as would be expected[15] from the fact that the dominant mouse and rat cone opsin, Opn1sw, has a λ_{max} of 360 nm. As the rat lens is 4 mm in axial length, while that of the mouse is ~ 2 mm, we used Beer's Law to adjust for the difference in length. Because the resultant spectrum has a transmission coefficient greater than 97% above 425 nm (the lowermost wavelength of reliability of our reflectance data – Fig. 6), for simplicity we set $D_{lens}(\lambda) = 0$ in the model calculations, but for completeness retained the symbol in the equations to follow. Rhodopsin absorption only applies to the dark adapted state; in the fully bleached state $D_{Rho} = 0$. The spectral form of $D_{mela}(\lambda)$ was that used by Kraats *et al.*[9], but scaled by a free (i.e., to-be- estimated) scale-factor Sf_{mela}. $D_{blood}(\lambda)$ was assumed to have the extinction spectrum of oxygenated hemoglobin, $\varepsilon_{HbO2}(\lambda)$, as tabulated by Scott Prahl (http://omlc.org/spectra/hemoglobin/summary.html), so that in the model $D_{blood}(\lambda) = Sf_{blood}\,\varepsilon_{HbO2}(\lambda)$, where Sf_{blood} is a free parameter. By Beer's Law $Sf_{blood} = C_{HbO2}\,d_{choroid}$, where C_{HbO2} is the average molar concentration of HbO_2 in the choroidal layer and $d_{choroid}$ its thickness (~ 45×10^{-4} cm; cf **Fig. 5C**). $D_{Rho}(\lambda)$ was derived from a mouse rhodopsin extinction spectrum (λ_{max} = 498 nm) calculated with a Lamb-Govardovskii template[4, 5] adjusted for an end-on axial absorbance of rhodopsin *in situ* $OD_{max} = 0.35$ (**Fig. 1D**). The "1-pass" spectral density functions obtained from the fitting of the model to the reflectance data are provided in **Fig. 6C**. (Here "1-pass" refers to propagation of light through a single layer with the respective density function: thus, for rhodopsin, one pass through a layer of the thickness of the rod outer segment; and for hemoglobin one pass through a 45 μm thick choroid.)

Reflective components. As illustrated by the OCT data (**Fig.5A, B**), back-scattering can occur from many fundus layers, with the most potent reflectance in the posterior eye. In the NIR OCT data, back-scattering from pre-outer segment layers has discrete peaks at the NFL, OPL and ELM, but as these show no obvious "bleach-dependence" we lumped the reflectance from all pre-outer segment elements into one

spectrally neutral parameter, $\rho_{pre\text{-}PR}$. As suggested by the OCT data, back-reflectance from rods was assumed to occur from two specific axial sites: the outer segment base or IS/OS junction ($\rho_{IS/OS}$), the outer segment tip (ρ_{tip}). (A weak reflectance distributed throughout the outer segment that was included in cone reflectance model of van de Kraats *et al.*[9] was not used in the modeling.) In keeping with the model of van de Kraats *et al.* the only reflecting layer deeper than the photoreceptors was assumed to be the sclera, with reflectance distribution[9] given by

$$\rho_{sclera}(\lambda) = \rho_{sclera}(675)\exp[-0.00261(\lambda - 675)] \qquad \text{Eq M1}$$

In modeling ρ_{sclera} (675) was taken as a free parameter to be estimated. (Although our reflectance data only extend to ~ 650 nm, we nonetheless elected to use ρ_{sclera} (675) as the free parameter for consistency with the van de Kraats et al. formulation.)

Scattering losses. Spectrally neutral scattering losses of incoming and reflected light were assumed to occur in two layers or media ("med"): the pre-outer segment retina ($D_{pre\text{-}PR}$) and the "deep" layers, including RPE and choroid (D_{deep}).

Bleaching-induced increment in rod reflectances. A unique feature of the mouse model was the inclusion of a factor for increased back-scattering at the rod IS/OS junction or OS base, and at the rod tips, a feature rationalize by OCT data (**Fig. 5**). Thus, the reflectances of the rod base and tip were assumed to be $\rho_{IS/OS}$ and ρ_{tip} in the dark adapted state, and $(1+Sf_{Blinc}) \times \rho_{IS/OS}$ and $(1+Sf_{Blinc}) \times \rho_{tip}$ in the fully bleached state, with $Sf_{Blinc} \geq 0$ estimated by fitting the model to the reflectance data. The inclusion of this factor was motivated by both OCT data (**Fig. 5C, D**), and the spectral reflectance data in the long wavelength portion of the spectrum, where rhodopsin absorption is negligible, and thus in which reflectance increases cannot arise from loss of light absorption by rhodopsin bleaching. (**Fig. 6A, B**). (The term "bleaching-induced" was adopted to identify photoactivation of rhodopsin, rather than light in general, as the cause of the increased reflectance. In this context "bleaching" physically equivalent to isomerization of the 11-*cis* chromophore of rhodopsin and its structural change to the enzymatically active Metarhodopsin II (λ_{max} = 380 nm), well established to be the form of the protein that activates phototransduction[16].) We have treated these $\rho_{IS/OS}$ and ρ_{tip} as spectrally flat

and equal for simplicity, but emphasize that they could have spectral dependence – e.g., if Raleigh scattering was involved, and need not be equal.

Mathematical formulation of the model.

In the following description wavelength-dependent factors such as density spectra are indicated as functions of λ, e.g., $D_{Rho}(\lambda)$. When not so indicated, the factors are assumed to be spectrally flat, i.e., have a single value not dependent on λ.

Pre-rod outer segment layers. Transmission of reflected light through the layers anterior to the rod IS/OS junction is given by

$$Trans_{PrePR}(\lambda) = 10^{-2[D_{lens}(\lambda)+D_{prePR}]} \qquad \text{Eq M2}$$

The factor 2 is present because light passes through the pre-photoreceptor media once on its incoming path and again on its outgoing path. Reflectance from pre-photoreceptor layers is treated as occurring at a single depth with a reflectivity ρ_{prePR} after one-way transmission through the pre-photoreceptor layers.

Rod layer. Reflectance at the rod outer segment base or IS/OS junctions and at the rod tips are described by

$$\begin{aligned} R_{IS/OS} &= [1+Sf_{Blinc}(1-p)]\times\rho_{IS/OS} \\ R_{tip} &= [1+Sf_{Blinc}(1-p)]\times\rho_{tip}\times 10^{-2[OD_{\max}\bar{\alpha}(\lambda)p]} \end{aligned} \qquad \text{Eq M3}$$

In the second line of Eq M3, $\bar{\alpha}(\lambda)$ represents the normalized Lamb-Govardovskii mouse rhodopsin extinction template, and p is the fraction rhodopsin present (1 for the dark adapted state; 0 for the fully bleached state). As previously noted, for simplification we omitted a weak, diffuse back reflection by the discs that is included in the van de Kraats *et al.*[12, 13] model. We assumed that a large fraction (>95%) of light propagating normal to the retina surface and reaching the IS layer is trapped in photoreceptor waveguides, with guiding beginning in the IS layer[17] where the photoreceptor cross sections tightly tile the retina. Let "Cov_{rods}" represent the coverage of the rods, i.e., the fraction of light reaching captured and guided by rods. The total light back reflected by rods is then given by

$$R_{rod,tot}(\lambda) = Cov_{rods}\times[R_{IS/OS}+R_{tip}(\lambda)] \qquad \text{Eq M4}$$

Cones constitute only 3% of the photoreceptors of the mouse retina, and although their inner segments are larger than those of rods, their outer segments are considerably smaller in width and length, and in the middle portion of the retina typically express only 10% or less M-opsin[18, 19] whose λ_{max}, 508 nm, is close to that of mouse rhodopsin. Thus, their contribution to the midwave light absorption in the dark adapted central retina is predictably only a few percent of that by rhodopsin, and can be neglected. It follows that the forward-propagating light transmitted past the outer segment layer is given by

$$Trans_{\mathrm{Post}PR}(\lambda) = (1 - Cov_{rods}) + (Cov_{rods})(1 - R_{IS/OS})[1 - R_{tip}(\lambda)] \times 10^{-OD_{\max}\bar{\alpha}(\lambda)p} \quad \text{Eq M5}$$

Post-receptor layers. In keeping with the van de Kraats *et al*. formulation, the model assumes that all reflection from post-receptor layers is of light that passes through the RPE and choroid and is back-reflected by the sclera. This "deep" reflectance is given by

$$R_{deep}(\lambda) = \rho_{sclera}(\lambda) \times 10^{-2[D_{mela}(\lambda) + D_{blood}(\lambda) + D_{deep}]} \quad \text{Eq M6}$$

Total reflection from the eye. The total reflectance of the eye combines the various terms described above as follows:

$$R_{eye}(\lambda) = Sf_{confocal} \times Trans_{\mathrm{Pre}PR}(\lambda) \times [\rho_{\mathrm{Pre}PR} + R_{rod,tot}(\lambda) + Trans_{PostPR}(\lambda) R_{deep}(\lambda)] \quad \text{Eq M7}$$

The absolute magnitudes of the reflectances found for the mouse eye (**Fig. 6**) are notably lower than those obtained in human studies[11, 12]. A key instrumental difference between the human reflectometry studies and ours of mice is that to deliver and collect light we employed a scanning laser ophthalmoscope, which acts confocally and thus restricts light collection to the limiting aperture of the imaging system[20]. We thus introduced a "confocal attenuation scale factor", Sf_{confocal}, which scales the entire predicted reflectance function (Eq M7).

Summary. In the simplest terms, the ocular reflectance model is basically "bookkeeping" of the total light budget entering and back-reflected from the eye. It keeps track of light of each wavelength as it propagates forward and is attenuated by absorption and scattering through the eye toward the deepest layers, and it cumulates the light back-reflected from three principal reflecting elements -- the pre-outer segment layers (R_{PrePR}), the rod base and tips ($R_{\mathrm{rod,tot}}$) and the sclera (R_{deep}) – as it makes the return

trip through the layers. The model incorporates several well known wavelength-dependent absorption factors in the different fundus layers, including rhodopsin in the outer segment layer, melanin in the RPE and choroid layers (pigmented mice), and oxygenated hemoglobin in the choroid layer. It also incorporates a spectral dependence reflectance function for the sclera (Eq M1).

Fitting the model to the reflectance data

The model was fitted to the spectral reflectance data of dark adapted and fully bleached albino and pigmented mice (**Fig. 6**) by least-squares minimization of a "chisquare" function:

$$\chi^2 = \sum_{i=1}^{2} \sum_{\lambda} \left[\frac{[R_{\text{eye,obs},i}(\lambda) - R_{\text{eye,model},i}(\lambda)]}{R_{\text{eye,model},i}(\lambda)} \right]^2 \qquad \text{Eq M7}$$

Here $i = 1, 2$ refer to the dark adapted and fully bleached states. Minimization was effected with a Matlab™ script employing the Nelder-Meade "fminsearch" algorithm. The values of eight parameters were extracted by searching over a continuous range (Table 2): ρ_{prePR}, D_{prePR}, D_{deep}, Sf_{Blinc}, Sf_{mela}, Sf_{blood}, ρ_{sclera} (675), Sf_{confocal}. The values of $\rho_{\text{IS/OS}}$, ρ_{tip} were chosen from fitting with a set of discrete values, maintaining $\rho_{\text{IS/OS}} = \rho_{\text{tip}}$. The end-on density of rhodopsin, OD_{max}, was fixed at a value (0.35) estimated from independent observations in the literature. We attempted to maintain the identical values of key parameters between the two strains of mice with the obvious exception that melanin density was set to zero ($Sf_{\text{mela}} = 0$) in fitting the albino mouse reflectance data. The almost 10-fold lower absolute reflectance of the C57Bl/6J mice, however, could not be accomodated without greater overall spectrally neutral attenuation, as expressed in the lower value of Sf_{confocal} and higher value of D_{prePR} for the model describing the pigmented mouse data (**Table S1**).

Though the model is necessarily complex and the parameter value combinations that generate reasonable descriptions of the data not unique, the model nonetheless serves to explain (i) a number of distinctive qualitative features of the reflectance data (**Fig. 6**), (ii) the dominant role of rhodopsin absorption loss in light-induced increases in reflectance in the middle wavelength portion of the spectrum (**Fig. 7**), and (iii) the necessary and sufficient role of including bleaching-induced increments in the reflectance

from the IS/OS junctions and OS tips of rods in explaining the change in fundus reflectance consequent to bleaching. The model also provides a useful means of testing hypotheses about the role of specific absorbing and reflecting elements in shaping the reflectance spectra, and provides a platform for generalizations that incorporate additional physical components. For example, we implemented model variants that included Bruch's membrane reflectance and melanin distributed in different deep compartments in C57Bl/6J, but found adding these additional complexities did little to improve the quality of the fits of the model, while adding additional free parameters. Comparison of the OCT depth profile data of albino and pigment mice (**Fig. 5**) suggests that melanin back-scattering plays a key role in C57Bl/6J mice, but as the wavelength dependence of this scattering has not been determined, we did not attempt to incorporate it. We acknowledge that the reflectance model is very much "a work in progress", but think that further refinements that incorporate insights from OCT, and that apply to data of both albino and pigmented mice (including mice with varied levels of pigmentation) hold much promise for extending the information obtained from mouse fundus imaging.

REFERENCES

1. Schmucker C, Schaeffel F. A paraxial schematic eye model for the growing C57BL/6 mouse. *Vision Research* 2004;44:1857-1867.
2. Remtulla S, Hallett PE. A schematic eye for the mouse, and comparisons with the rat. *Vision Research* 1985;25:21-31.
3. Geng Y, Schery LA, Sharma R, et al. Optical properties of the mouse eye. *Biomedical Optics express* 2011;2:717-738.
4. Lamb TD. Photoreceptor spectral sensitivities: common shape in the long-wavelength region. *Vision Research* 1995;35:3083-3091.
5. Govardovskii VI, Fyhrquist N, Reuter T, Kuzmin DG, Donner K. In search of the visual pigment template. *Visual Neuroscience* 2000;17:509-528.
6. Liebman PA. In situ microspectrophotometric studies on the pigments of single retinal rods. *Biophysical Journal* 1962;2:161-178.

7. Liebman PA. Microspectrophotometry of photoreceptors. In: Dartnall HJA (ed), *Photochemistry of Vision.* Berlin: Springer- Verlag; 1972:481-528.
8. Harosi FI. Cynomolgus and rhesus monkey visual pigments. Application of Fourier transform smoothing and statistical techniques to the determination of spectral parameters. *Journal of General Physiology* 1987;89:717-743.
9. Lyubarsky AL, Daniele LL, Pugh EN, Jr. From candelas to photoisomerizations in the mouse eye by rhodopsin bleaching in situ and the light-rearing dependence of the major components of the mouse ERG. *Vision Research* 2004;44:3235-3251.
10. Carter-Dawson LD, LaVail MM. Rods and cones in the mouse retina. I. Structural analysis using light and electron microscopy. *Journal of Comparative Neurology* 1979;188:245-262.
11. Delori FC, Pflibsen KP. Spectral reflectance of the human ocular fundus. *Applied Optics* 1989;28:1061-1077.
12. van de Kraats J, Berendschot TTJM, Van Norren D. The pathways of light measured in fundus reflectometry. *Vision Research* 1996;36:2229-2247.
13. Van Norren D, Van de Kraats J. Imaging retinal densitometry with a confocal scanning laser ophthalmoscope. *Vision Research* 1989;29:1825-1830.
14. Morgan JI, Pugh EN, Jr. Scanning laser ophthalmoscope measurement of local fundus reflectance and autofluorescence changes arising from rhodopsin bleaching and regeneration. *Investigative Ophthalmology & Visual science* 2013;54:2048-2059.
15. Douglas RH, Jeffery G. The spectral transmission of ocular media suggests ultraviolet sensitivity is widespread among mammals. *Proceedings Biological sciences / The Royal Society* 2014;281:20132995.
16. Choe HW, Kim YJ, Park JH, et al. Crystal structure of metarhodopsin II. *Nature* 2011;471:651-655.
17. Snyder AW, Pask C. The Stiles-Crawford effect--explanation and consequences. *Vision research* 1973;13:1115-1137.

18. Daniele LL, Insinna C, Chance R, Wang J, Nikonov SS, Pugh EN, Jr. A mouse M-opsin monochromat: retinal cone photoreceptors have increased M-opsin expression when S-opsin is knocked out. *Vision research* 2011;51:447-458.

19. Nikonov SS, Kholodenko R, Lem J, Pugh EN, Jr. Physiological features of the S- and M-cone photoreceptors of wild-type mice from single-cell recordings. *The Journal of general physiology* 2006;127:359-374.

20. Zhang P, Goswami M, Zam A, Pugh EN, Zawadzki RJ. Effect of scanning beam size on the lateral resolution of mouse retinal imaging with SLO. *Optics letters* 2015;40:5830-5833.

Table S1. Parameters of the Reflectance Model

Parameter description	Symbol	Balb/c value	C57Bl/6J value	Balb/c range	C57Bl/6J range	Comment
Scattering density of pre-ELM media	D_{prePR}	**0.0**	**0.39**	0 – 0.05	0.2 – 0.5	Notably higher for C57Bl/6J
Reflectance of pre-ELM media	ρ_{prePR}	**0.007**	**0.051**	0.005 – 0.15	0.02 – 0.6	Notably higher for C57Bl/6J
Scattering density of post-photoreceptor media	D_{deep}	**0.073**	**0.0**	0.03 – 0.10	NA	Low value in C57Bl/6 likely reflects dominance of melanin
Scale factor for scleral reflectance spectrum	$\rho_{\text{sclera}}(675)$	**0.061**	**0.140**	0.03 – 0.10	0.05 – 0.2	C57Bl/6J data suggest that melanin backscattering plays a role in pigmented mice.
Scale factor for melanin absorption spectrum	Sf_{mela}	**0.0**	**2.18**	NA	1.8 – 2.8	$D_{\text{mela}}(\lambda)= Sf_{\text{mela}}\, Abs_{\text{mela}}(\lambda)$; $Abs_{\text{mela}}(500) = 0.40$
Avg. $[HbO_2]$ in choroid	C_{HbO2} (mM)	**2.0**	**2.0**	1.8 – 2.2	NA	$D_{\text{blood}}(\lambda) = \varepsilon_{\text{HbO2}}(\lambda)\, C_{\text{HbO2}}\, d_{\text{choroid}}$, $d_{\text{choroid}} = 45\ (30) \times 10^{-4}$ cm in Balbc (C57Bl/6J)
Scale factor for photoreceptor “Blinc”	Sf_{Blinc}	**0.35**	**0.35**	0.3 – 0.7	0.2 – 0.4	Explains non-rhodopsin component of incremental reflectance in visible & NIR (Figs. 6, 7)
Scale factor for “confocal attenuation”	Sf_{confocal}	**0.246**	**0.088**	0.15 – 0.27	0.05 – 0.1	Notably lower in C57Bl/6J
Reflectance of IS/OS junction	$\rho_{\text{IS/OS}}$	**0.001**	**0.02**	0.02 – 0.05	0.02 – 0.05	Motivated by OCT data (Fig. 5); necessary and sufficient for good fitting of model (Fig. 6)
Reflectance of rod tips	ρ_{tip}	**0.001**	**0.02**	0.02 – 0.05	0.02 – 0.05	Motivated by OCT data (Fig. 5); necessary and sufficient for good fitting of model (Fig. 6)

Table notes. The first two columns present the description and symbolic identification of a parameter used in the fundus spectral reflectance model (Supplementary Material IV). The third and fourth columns present the values of the parameters in the versions of the model fitting Balb/c (**Fig. 6A**) and C57Bl/6J (**Fig. 6B**). The fifth and six columns present ranges of parameter values for which "good fits" of the model to the data of each strain could be obtained when all other parameters were free to vary. ("Good fit" means that the Chisq value of the fit was within ~ 15% of the minimum.) All the factors in the table with the exception of $\rho_{sclera}(675)$ are spectrally neutral. The spectral dependence of the reflectance model arises from the absorption of light by the rhodopsin, melanin and oxygenated hemoglobin and from the spectral dependence of the scleral reflectance, as explained in the presentation of the model (see **Fig. 6C**).
